# Supplementary material for: Developmental Gene Expression Differences between Humans and Mammalian Models
Source: Cell Rep. 2020 Oct 27;33(4):108308. doi: 10.1016/j.celrep.2020.108308 (PMC7610014; doi:10.1016/j.celrep.2020.108308)
Supplement: Document S1. Figures S1–S7 [file mmc1.pdf]

Cell Reports, Volume *33*

Supplemental Information

Developmental Gene Expression Differences

between Humans and Mammalian Models

Margarida Cardoso-Moreira, Ioannis Sarropoulos, Britta Velten, Matthew Mort, David N. Cooper, Wolfgang Huber, and Henrik Kaessmann

Figure S1

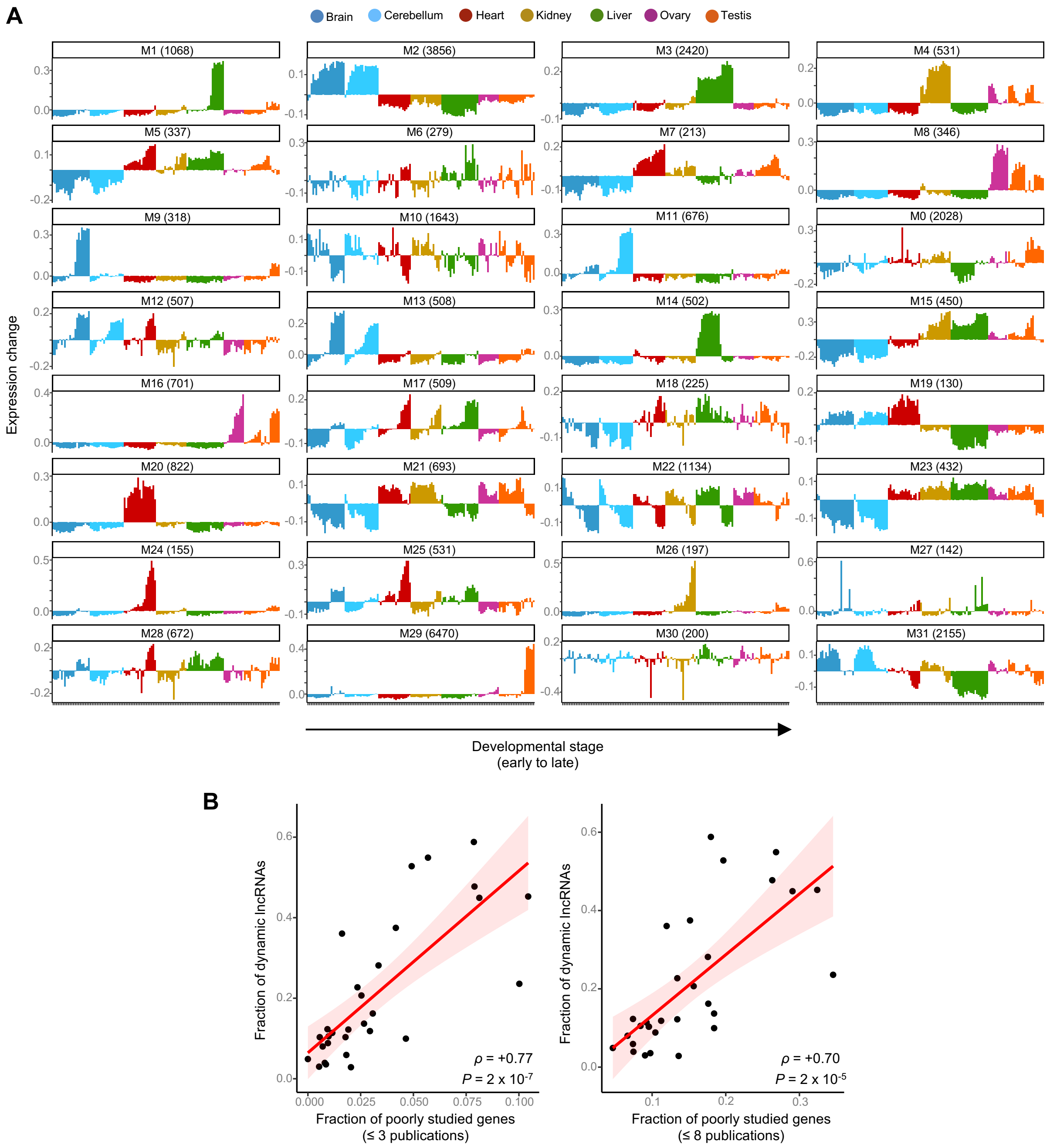

Figure S1. Human weighted gene co-expression network, Related to Figure 1 (A) Organ developmental profiles for each module; shown is the module's eigengene. **(B)** There is a strong positive correlation between the fraction of developmentally dynamic lncRNAs in a module and the fraction of poorly studied protein-coding genes. Poorly studied genes are those with 3 or fewer publications (left) or those with 8 or fewer publications (right). Data on the number of publications are from Stoeger and colleagues (Stoeger et al., 2018). Shaded area corresponds to the 95% confidence interval.

Figure S2

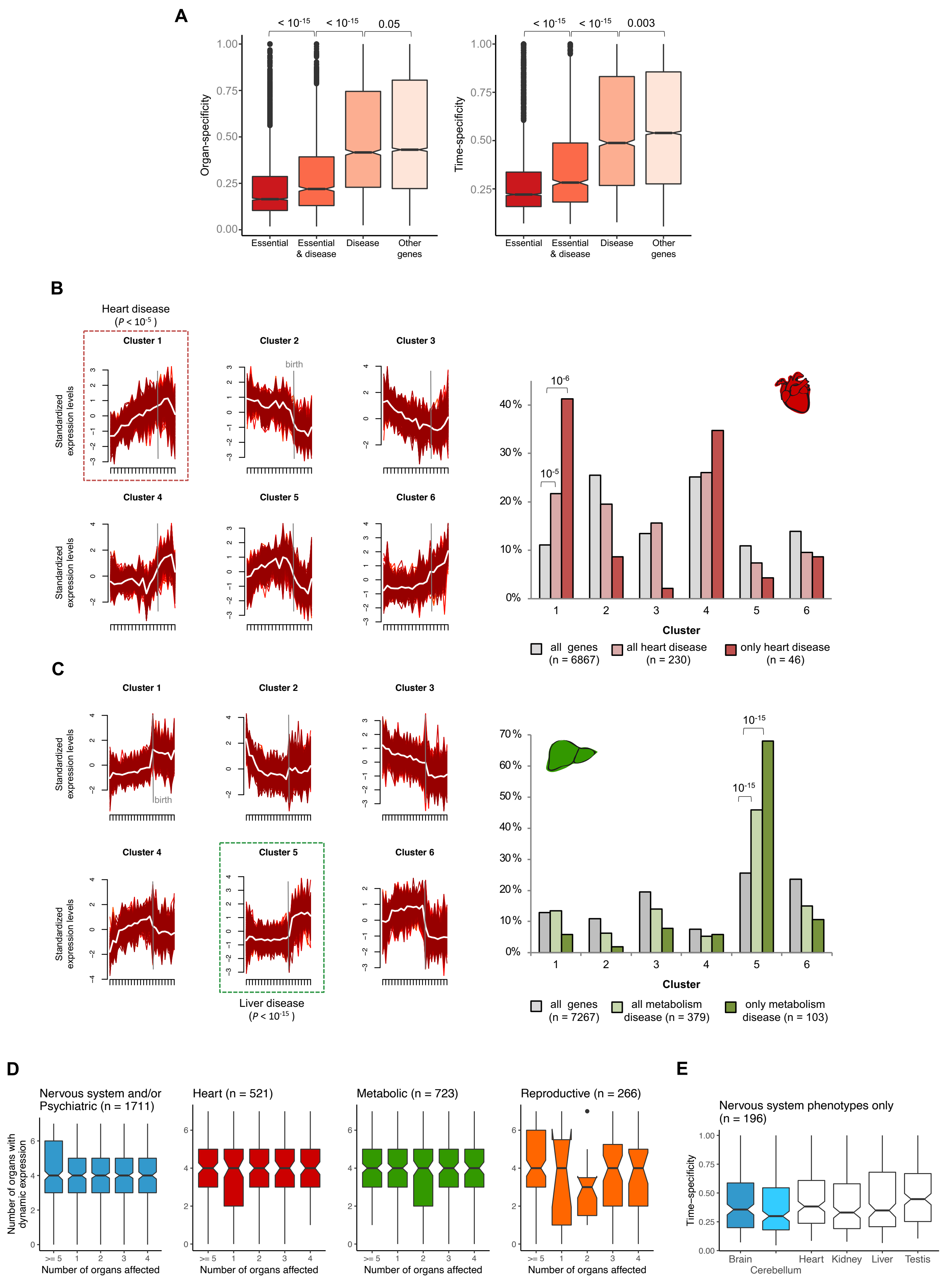

A
< 10-15
< 10-15
0.05
Organ-specificity
Essential
Essential & disease
Disease
Other genes
< 10-15
< 10-15
0.003
Time-specificity
B
Heart disease (P < 10-5)
Cluster 1
Cluster 2
Cluster 3
Cluster 4
Cluster 5
Cluster 6
birth
Standardized expression levels
10-6
10-5
Cluster
all genes (n = 6867)
all heart disease (n = 230)
only heart disease (n = 46)
C
Cluster 1
Cluster 2
Cluster 3
Cluster 4
Cluster 5
Cluster 6
Liver disease (P < 10-15)
10-15
10-15
Cluster
all genes (n = 7267)
all metabolism disease (n = 379)
only metabolism disease (n = 103)
D
Nervous system and/or Psychiatric (n = 1711)
Heart (n = 521)
Metabolic (n = 723)
Reproductive (n = 266)
Number of organs with dynamic expression
Number of organs affected
E
Nervous system phenotypes only (n = 196)
Time-specificity
Brain
Cerebellum
Heart
Kidney
Liver
Testis

Figure S2. Spatiotemporal profiles of disease genes, Related to Figure 2. (A) Organ- and time-specificity (median across organs) for genes in different classes of phenotypic severity (*P*-values from Wilcoxon rank sum test, two-sided). The box plots depict the median ± 25th and 75th percentiles, whiskers at 1.5 times the interquartile range. The number of genes in each class is provided in Figure 2A. **(B)** Distribution of genes associated with heart disease among the 6 heart clusters. The developmental profiles of the 6 clusters are shown on the left. The y-axis shows standardized expression levels (Methods). The profile of each gene in the cluster is shown in red; the white line shows the cluster center. Cluster 1 is enriched for heart disease-associated genes both when using all genes associated with a heart phenotype (50 out of 230 genes) and when restricting the set to those exclusively associated with the heart (19 out of 46 genes) (*P*-values from binomial tests after Bonferroni correction). **(C)** Distribution of genes associated with metabolic diseases among the 6 liver clusters. The developmental profiles of the 6 clusters are shown on the left. The y-axis shows standardized expression levels (Methods). The profile of each gene in the cluster is shown in red; the white line shows the cluster centre. Cluster 5 is enriched for metabolic disease-associated genes both when using all genes associated with a metabolic phenotype (174 out of 379 genes) and when restricting the set to those exclusively associated with metabolism (70 out of 103 genes) (*P*-values from binomial tests after Bonferroni correction). **(D)** Number of organs where genes have dynamic temporal profiles as a function of the number of organs where they are known to cause disease. Number of genes in each disease class in parenthesis. **(E)** Time-specificity in different organs for genes associated exclusively with nervous system phenotypes (n = 196 genes). In **(D)** and **(E)** the box plots depict the median ± 25th and 75th percentiles, whiskers at 1.5 times the interquartile range.

Figure S3

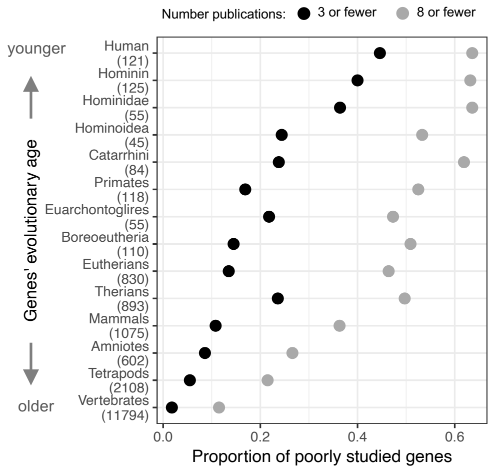

Figure S3: Recently originated genes tend to be poorly studied, Related to Figure 3. The proportion of poorly studied genes (i.e., those with 3 or fewer publications (black) or those with 8 or fewer publications (grey)) increases for genes of successively younger evolutionary ages (i.e. with more recent origins). In this analysis, the youngest genes are those that are human-specific (top) and the oldest are those shared across vertebrates (bottom). In parenthesis are the number of genes in each age class. Data on the evolutionary age of genes are from GenTree (Shao et al., 2019) and data on the number of publications are from Stoeger and colleagues (Stoeger et al., 2018). The dataset of Stoeger and colleagues does not include data for most recently originated genes (those originated along the primate lineage), so the estimates provided are conservative (i.e., current knowledge on these genes is even more limited).

Figure S4

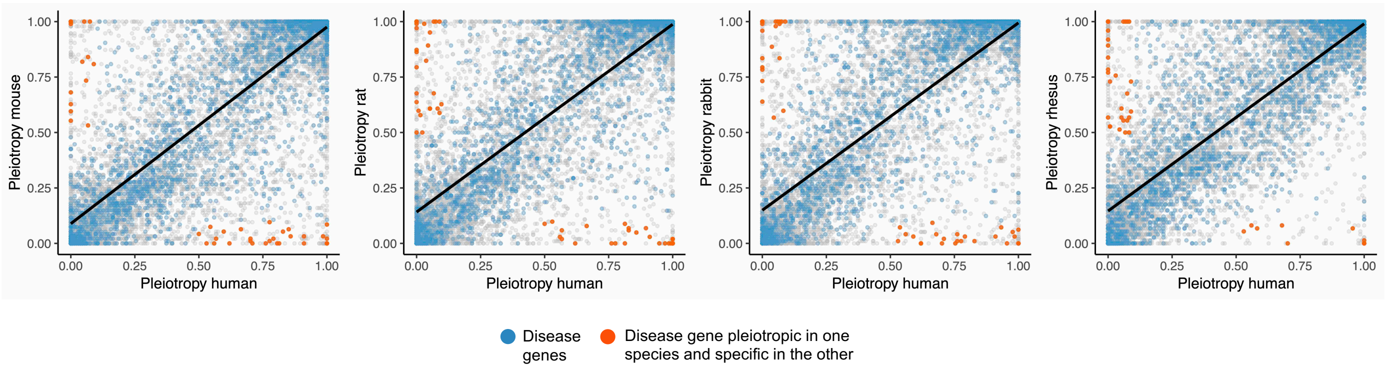

Figure S4: Breadth of spatiotemporal expression of human genes and their orthologs in mouse, rat, rabbit and rhesus, Related to Figure 4. Relationship between human expression pleiotropy and those of the other species. The blue dots denote disease-associated genes and the orange dots denote disease-associated genes expressed in at least 50% of the samples in one species but in less than 10% of the samples in the other. Note that the rhesus macaque time series is shorter, not covering the period of embryonic development sampled in the other species.

Figure S5

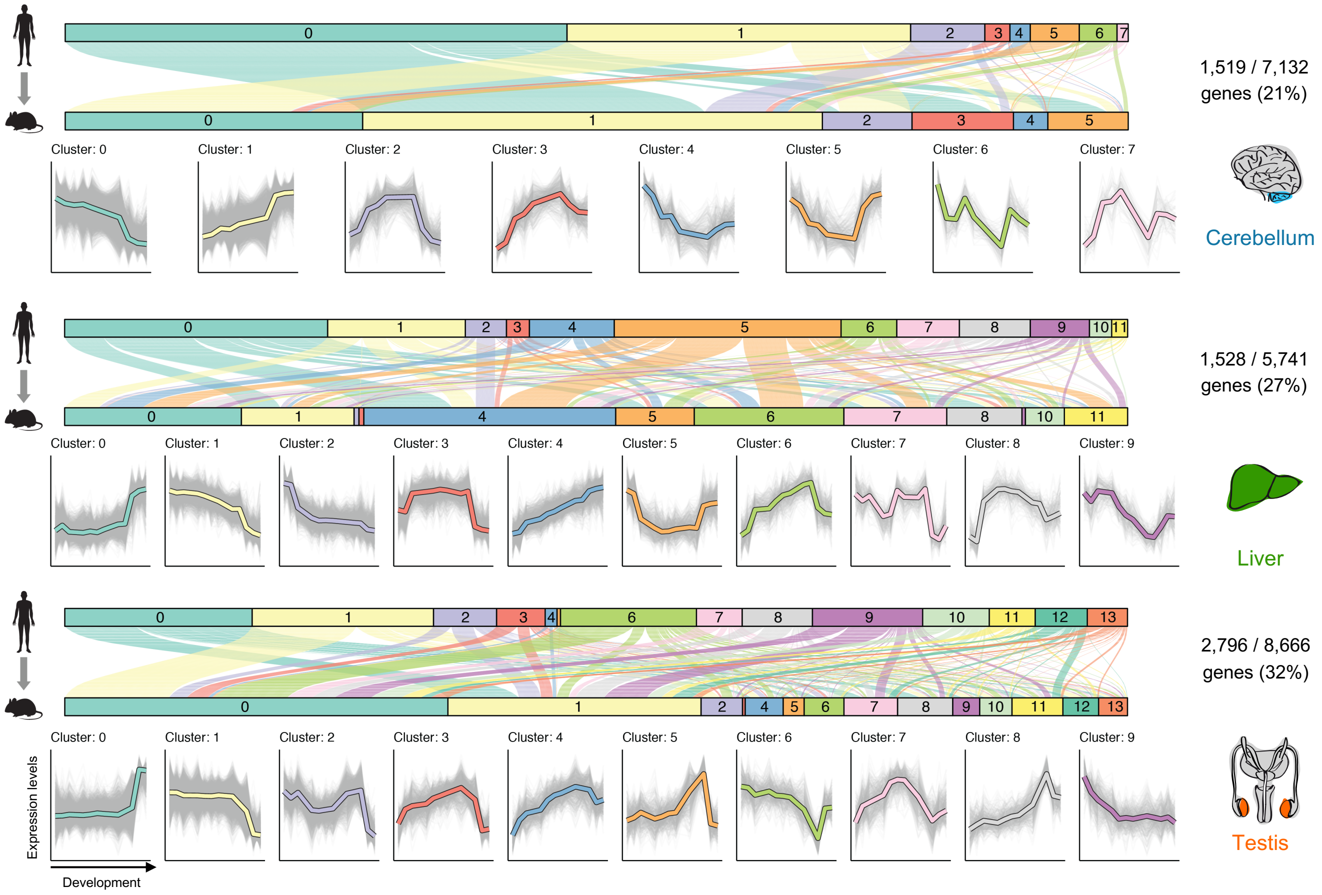

Figure S5: Developmental trajectory differences between human and mouse in cerebellum, liver and testis, Related to Figure 5. We used soft clustering to group human genes and their mouse orthologs according to their temporal expression in each organ. The genes for which the human and mouse ortholog were assigned to a different cluster are shown on top. Each line represents a pair of human-mouse orthologs and shows the cluster assignment in human (top) and mouse (bottom). The lines are colored according to the human cluster assignment. The clusters identified in each organ are shown below (grey lines correspond to individual genes and the colored lines to the cluster center, which is akin to the median expression of the cluster). The y-axis shows the log normalized expression levels and the x-axis shows the samples ordered from early to late development. In liver and testis, only the profiles of the first 9 clusters are shown. This panel complements Figure 5 which shows the same analysis for brain, heart and kidney.

Figure S6

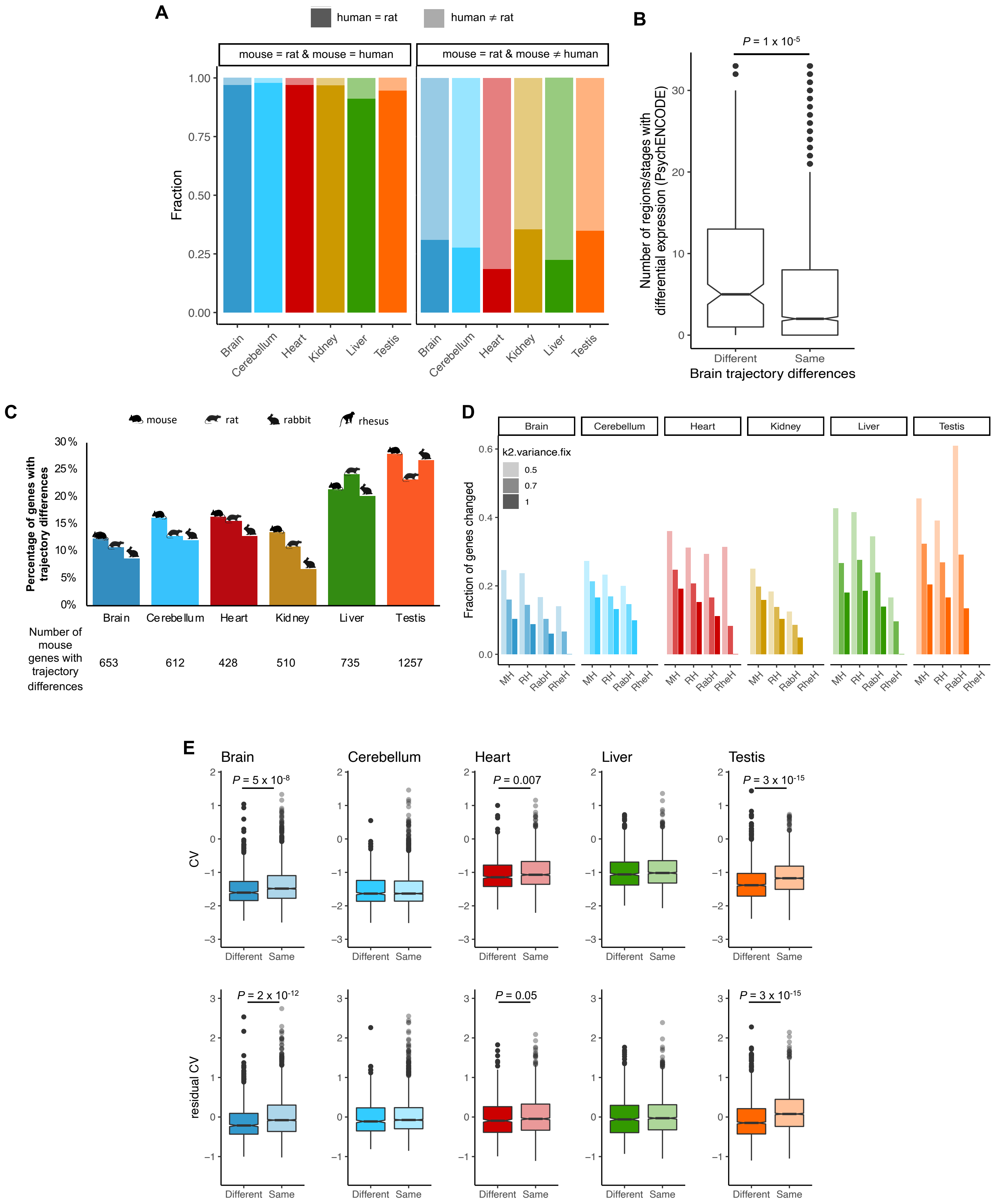
A
human = rat
human ≠ rat
mouse = rat & mouse = human
mouse = rat & mouse ≠ human
Fraction
Brain
Cerebellum
Heart
Kidney
Liver
Testis
B
P = 1 x 10-5
Number of regions/stages with differential expression (PsychENCODE)
Different
Same
Brain trajectory differences
C
mouse
rat
rabbit
rhesus
Percentage of genes with trajectory differences
Number of mouse genes with trajectory differences
653
612
428
510
735
1257
D
k2.variance.fix
Fraction of genes changed
MH
RH
RabH
RheH
E
CV
residual CV
P = 5 x 10-8
P = 0.007
P = 3 x 10-15
P = 2 x 10-12
P = 0.05
P = 3 x 10-15

Figure S6: Developmental trajectory differences between human and the other species, Related to Figure 6. (A) If a gene has a similar trajectory between mouse and rat, then the human-mouse comparison is expected to give a similar result to the human-rat comparison. The panel on the left shows that most genes (96% across organs) called as having a similar trajectory between mouse and rat and between human and mouse, are also called as having a similar trajectory between human and rat (as expected). The panel on the right shows that most genes (70% across organs) called as having a similar trajectory between mouse and rat and a different trajectory between human and mouse, are also called as having a different trajectory between human and rat. **(B)** The PsychENCODE consortium compared gene expression profiles between human and rhesus macaque for 11 regions of the neocortex and for 3 developmental periods (i.e., prenatal, postnatal, and adult), for a total of 33 comparisons. We compared the genes that we identified as having similar or different brain developmental trajectories between human and rhesus macaque in terms of the number of comparisons that the PsychENCODE dataset called as differentially expressed. The PsychENCODE dataset provided comparisons for 234 of the 399 genes that we identified as having trajectory differences and for 2,327 of the 5,615 genes that we identified as having similar trajectories. The *P*-value is from a Wilcoxon rank sum test. The box plots depict the median ± 25th and 75th percentiles, whiskers at 1.5 times the interquartile range. **(C)** Percentage of genes in each organ that have different trajectories between human and mouse, rat and rabbit. This analysis is similar to that in Figure 6A except that the set of genes tested for trajectory differences is the same for all species (i.e., 5287 1:1 orthologous genes across the four species tested in the brain, 3786 in the cerebellum, 2608 in the heart, 3767 in the kidney, 3430 in the liver and 4500 in the testis). **(D)** Proportion of genes with different trajectories between human (H) and mouse (M), rat (R), rabbit (Rab) and Rhesus (Rhe) using different thresholds of k2.variance.fix (k = 1, k = 0.7 and k = 0.5). The set of genes identified using k=0.7 includes the set of genes identified using k=1; the set of genes identified using k=0.5 includes the sets of genes identified using k=1 and k=0.7. **(E)** Differences in gene expression variation among humans (GTEx dataset) between genes identified as having different or similar trajectories in the human-mouse comparison. The top row shows the variation of gene expression estimated as the coefficient of variation (CV; standard deviation divided by the mean) and the bottom row shows a derived measure of the coefficient of variation, residual CV, which also takes into consideration differences in gene expression levels (Methods). All *P*-values are from Wilcoxon rank sum tests after Bonferroni correction. The box plots depict the median ± 25th and 75th percentiles, whiskers at 1.5 times the interquartile range.

Figure S7

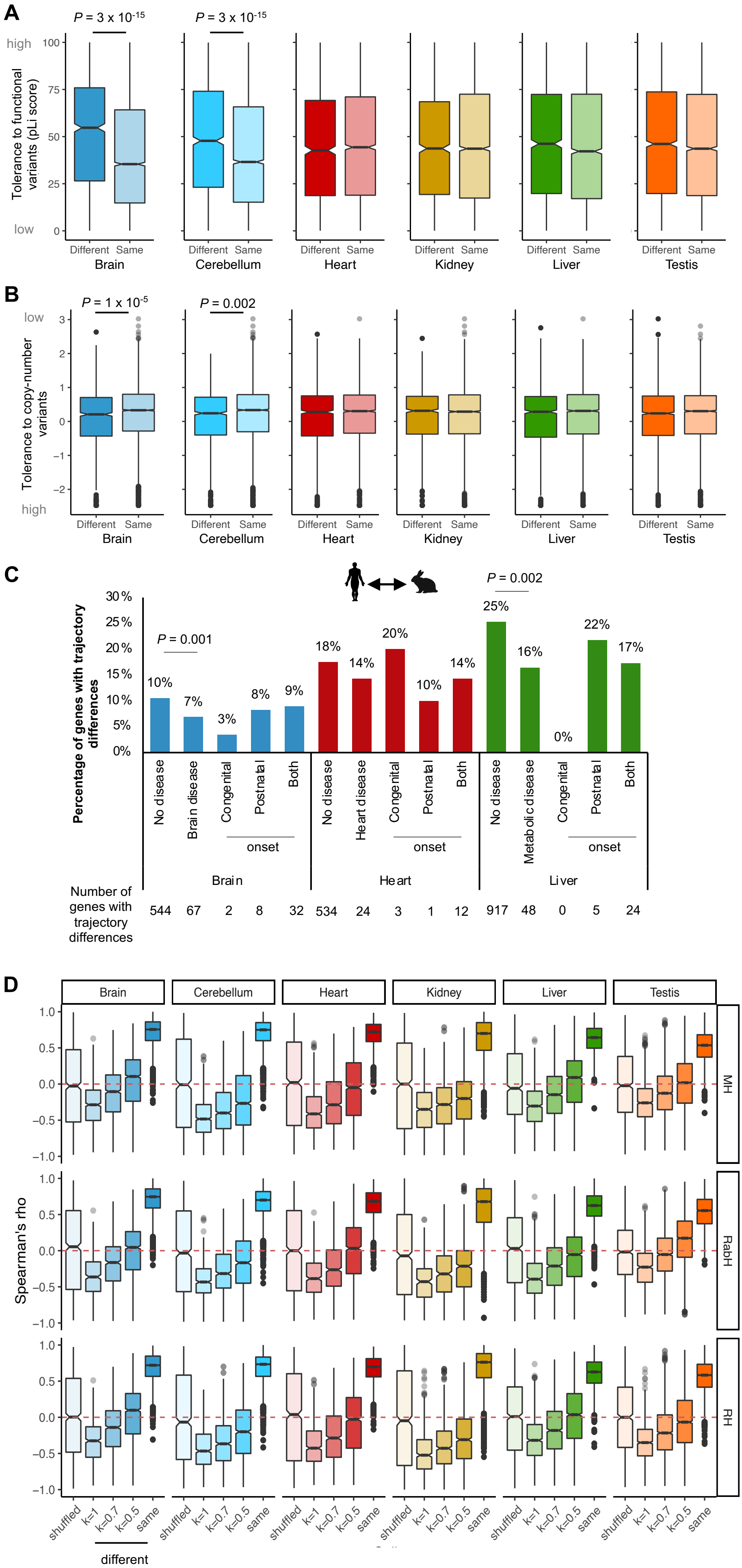

A
P = 3 x 10-15
P = 3 x 10-15
high
low
Tolerance to functional variants (pLi score)
100
75
50
25
0
Different Same
Brain
Cerebellum
Heart
Kidney
Liver
Testis
B
P = 1 x 10-5
P = 0.002
low
high
Tolerance to copy-number variants
3
2
1
0
−1
−2
C
P = 0.001
P = 0.002
Percentage of genes with trajectory differences
30%
25%
20%
15%
10%
5%
0%
10% 7% 3% 8% 9% 18% 14% 20% 10% 14% 25% 16% 0% 22% 17%
No disease
Brain disease
Heart disease
Metabolic disease
Congenital
Postnatal
Both
onset
Brain
Heart
Liver
Number of genes with trajectory differences
544 67 2 8 32 534 24 3 1 12 917 48 0 5 24
D
Brain
Cerebellum
Heart
Kidney
Liver
Testis
MH
RabH
RH
Spearman's rho
1.0
0.5
0.0
−0.5
−1.0
shuffled
k=1
k=0.7
k=0.5
same
different

Figure S7: Developmental trajectory differences between human and other species, Related to Figure 6. (A) Intolerance to functional mutations (probability that a gene is intolerant to a loss-of-function mutation, known as 'pLI' score) for genes with different or similar trajectories between human and mouse. Low values on the y-axis means low tolerance to functional mutations. **(B)** Intolerance to copy-number variants (probability that a gene is intolerant duplication and/or deletion polymorphisms) for genes with different or similar trajectories between human and mouse. Low values on the y-axis means high tolerance to copy-number variants. **(C)** Percentage of genes in brain, heart and liver that differ in trajectories between human and rabbit. Bonferroni-corrected *P*-values for comparisons between disease and non-disease genes are from Fisher's exact tests and Bonferroni-corrected *P*-values for comparisons of disease genes with different ages of onset are from binomial tests. **(D)** Spearman correlation of the temporal expression of human genes and their orthologs in mouse (top), rabbit (middle) and rat (bottom) for genes called as having different trajectories using different values of k2.variance.fix (k = 1, k = 0.7 and k = 0.5) and for genes called as having the same trajectory. Correlations for unrelated (non-orthologous) pairs of genes are also shown ("shuffled") as reference.
