# Supplementary material for: Developmental Gene Expression Differences between Humans and Mammalian Models
Source: Cell Rep. 2020 Oct 27;33(4):108308. doi: 10.1016/j.celrep.2020.108308 (PMC7610014; doi:10.1016/j.celrep.2020.108308)
Supplement: Document S2. Article plus Supplemental Information [file mmc7.pdf]

Cell Reports

Article

Developmental Gene Expression Differences between Humans and Mammalian Models

Graphical Abstract

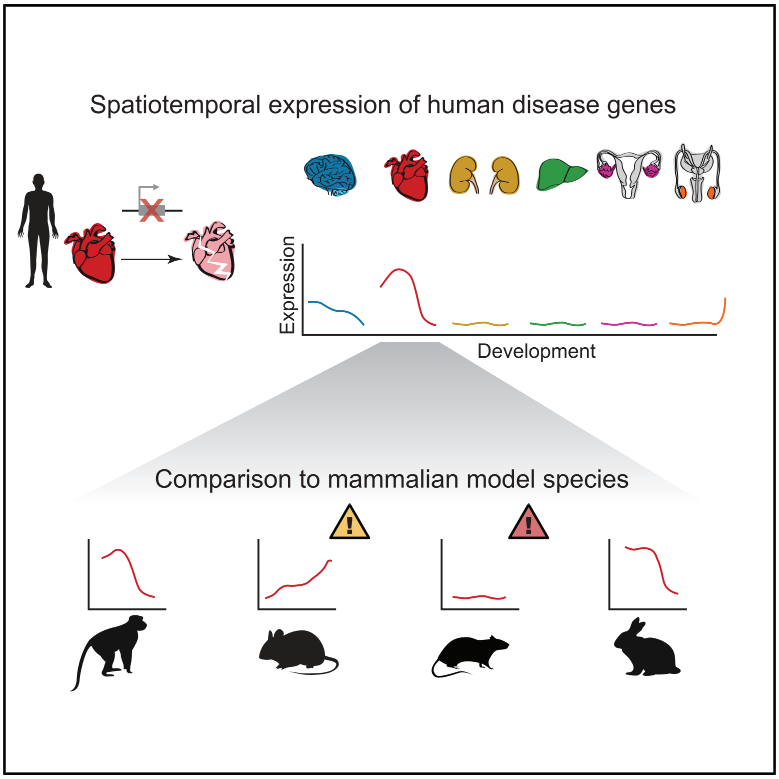

Highlights

- Associations between disease genes' developmental profiles and clinical phenotypes
- Comparison of human genes with their orthologs in four mammalian model species
- Half of human genes differ from mouse orthologs in organ temporal trajectories
- These include more than 200 genes associated with brain, heart, and liver disease

Authors

Margarida Cardoso-Moreira, Ioannis Sarropoulos, Britta Velten, Matthew Mort, David N. Cooper, Wolfgang Huber, Henrik Kaessmann

Correspondence

margarida.cardosomoreira@crick.ac.uk (M.C.-M.), h.kaessmann@zmbh.uni-heidelberg.de (H.K.)

In Brief

Cardoso-Moreira et al. identify associations between the developmental expression of disease genes and clinical phenotypes. They also compare the similarity of spatiotemporal expression between human genes and their orthologs in rhesus macaque, mouse, rat, and rabbit and find that organ temporal trajectory differences are common and include many disease genes.

Cardoso-Moreira et al., 2020, Cell Reports *33*, 108308
October 27, 2020 © 2020 The Author(s).
https://doi.org/10.1016/j.celrep.2020.108308

CellPress

Cell Reports

CellPress
OPEN ACCESS

Article

Developmental Gene Expression Differences between Humans and Mammalian Models

Margarida Cardoso-Moreira,[1,4,*] Ioannis Sarropoulos,[1] Britta Velten,[2] Matthew Mort,[3] David N. Cooper,[3] Wolfgang Huber,[2] and Henrik Kaessmann[1,*]
[1]Center for Molecular Biology (ZMBH), DKFZ-ZMBH Alliance, Heidelberg University, 69120 Heidelberg, Germany
[2]Genome Biology Unit, European Molecular Biology Laboratory, 69117 Heidelberg, Germany
[3]Institute of Medical Genetics, Cardiff University, Cardiff CF14 4XN, UK
[4]Lead Contact
*Correspondence: margarida.cardosomoreira@crick.ac.uk (M.C.-M.), h.kaessmann@zmbh.uni-heidelberg.de (H.K.)
https://doi.org/10.1016/j.celrep.2020.108308

SUMMARY

Identifying the molecular programs underlying human organ development and how they differ from model species is key for understanding human health and disease. Developmental gene expression profiles provide a window into the genes underlying organ development and a direct means to compare them across species. We use a transcriptomic resource covering the development of seven organs to characterize the temporal profiles of human genes associated with distinct disease classes and to determine, for each human gene, the similarity of its spatiotemporal expression with its orthologs in rhesus macaque, mouse, rat, and rabbit. We find clear associations between spatiotemporal profiles and the phenotypic manifestations of diseases. We also find that half of human genes differ from their mouse orthologs in their temporal trajectories in at least one of the organs. These include more than 200 genes associated with brain, heart, and liver disease for which mouse models should undergo extra scrutiny.

INTRODUCTION

The genetic programs underlying human organ development are only partially understood, yet they are fundamental to understanding organ morphology, physiology, and disease (Bruneau, 2013; DeFalco and Capel, 2009; Si-Tayeb et al., 2010; Silbereis et al., 2016; Vainio and Lin, 2002; Wang and Zoghbi, 2001). Gene expression is a molecular readout of developmental processes and therefore provides a window into the genes and regulatory networks underlying organ development (Lein et al., 2017; Pantalacci and Semon, 2014). By densely profiling gene expression throughout organ development, we get closer to identifying the genes and molecular processes underlying organ differentiation, maturation, and physiology (Bakken et al., 2016; Cardoso-Moreira et al., 2019; Giudice et al., 2014; Houmard et al., 2009; Zhu et al., 2018). In addition, spatiotemporal gene expression profiles provide a wealth of information on human disease genes, which can be leveraged to gain new insights into the etiology and symptomatology of diseases (Finucane et al., 2018; Gerrelli et al., 2015; Lein et al., 2017; Li et al., 2018).

Much of the progress made in identifying the genetic programs underlying human organ development has come from research in model organisms. Mice and other mammals (e.g., rats and rhesus macaques) are routinely used as models of normal human development and disease because it is generally assumed that the genetic programs underlying development are largely conserved across these species. While usually true, there are also critical differences between species during development, which underlie the large diversity of mammalian organ phenotypes (Bruneau, 2013; DeFalco and Capel, 2009; Lein et al., 2017; Si-tayeb et al., 2016; Silbereis et al., 2016; Vainio and Lin, 2002; Wang and Zoghbi, 2001). Identifying the commonalities and differences between the genetic programs underlying organ development in different mammalian species is therefore key for assessing the translatability of knowledge obtained from mammalian models to understand human health and disease. Critically, gene expression profiles can be directly compared between species, especially when they are derived from matching cells/organs and developmental stages. Although there are challenges (e.g., it is easier to compare gene expression for more closely related species, and comparisons are limited to genes with a 1:1 orthology relationship between species), gene expression offers a direct means to evaluate similarities and differences between species in organ developmental programs (reviewed in Pantalacci and Semon, 2014). While the relationship between gene expression and phenotypes is not linear, identifying when and where gene expression differs between humans and other species will help identify the conditions (i.e., developmental stages, organs, and genes) under which model species may not be well suited to model human development and disease.

To characterize the organ developmental profiles of human disease genes and gain new insights into the symptomatology of diseases, we use a developmental gene expression resource that we recently generated (Cardoso-Moreira et al., 2019). This dataset densely covers the development of seven major organs in humans and other mammals. For each human gene in our

This is an open access article under the CC BY license (http://creativecommons.org/licenses/by/4.0/).

dataset (including disease-associated genes), we determine the similarity of its spatiotemporal expression with that of its orthologs in mouse, rat, rabbit, and rhesus macaque, providing a new resource that is relevant for the choice of mammalian species to model the action of individual genes and/or processes in both healthy and pathological human organ development.

RESULTS

An Expression Atlas of Human Organ Development

This work is based on a resource that we recently generated (Cardoso-Moreira et al., 2019), which provides human gene expression time series for seven major organs: brain (forebrain/cerebrum), cerebellum (hindbrain/cerebellum), heart, kidney, liver, ovary, and testis (Figure 1A). The time series starts at 4 weeks post-conception (wpc), which corresponds to early organogenesis for all organs except the heart (mid-organogenesis), and then covers prenatal development weekly until 20 wpc. The sampling restarts at birth and spans major developmental milestones, including aging (Figure 1A; total of 297 RNA-sequencing [RNA-seq] libraries). This resource also provides matching datasets for four species commonly used to study human development and disease: mouse (316 libraries), rat (350 libraries), rabbit (315 libraries), and rhesus macaque (starting at a late fetal stage that corresponds to 19 wpc in human; 154 libraries; STAR Methods).

We used a weighted gene co-expression network analysis to identify the main clusters (modules) of highly correlated genes during human organ development (STAR Methods). We then characterized each module according to its developmental profile (Figures 1B and S1A), functional and disease enrichments (Figure 1B; Table S1), and proportion of transcription factors (TFs) (Zhang et al., 2015), RNA-binding proteins (RBPs) (Gerstberger et al., 2014), and developmentally dynamic long noncoding RNAs (lncRNAs) (Sarropoulos et al., 2019) (Figure 1B). As expected, we observed a match between the disease enrichments of each module and its organ developmental profile (Figure 1B). For example, module M3 comprises 2,420 genes predominantly expressed in the liver and is enriched for several liver-related diseases (e.g., fatty liver). Module M20 (822 genes) comprises genes mainly expressed in the heart and is associated with a number of cardiomyopathies.

Through ‘‘guilt by association’’, these modules additionally provide putative functions for poorly characterized genes (Table S2). Surprisingly, we identified a strong positive correlation between the fraction of protein-coding genes in a module that are among the least studied in the human genome (based on Stoeger et al., 2018) and the module’s fraction of dynamic lncRNAs (ρ: +0.77, p value = 2×10^{-7}; Figure S1B). Modules rich in poorly studied protein-coding genes and developmentally dynamic lncRNAs are frequently associated with high expression in the gonads (Figure 1B) but are also found in association with high expression in each of the other organs (e.g., module M9 for brain and module M11 for cerebellum).

Spatiotemporal Profiles of Disease Genes

We used this expression atlas of human organ development to test for associations between the spatiotemporal profiles of human disease genes and the etiology and phenotypic manifestation of human diseases. We first assigned genes to different classes of phenotypic severity by integrating a dataset of human essential genes (Bartha et al., 2018) with a dataset of genes associated with inherited disease in the manually curated Human Gene Mutation Database (‘‘disease genes’’) (Stenson et al., 2017) (Figure 2A). We then compared the breadth of developmental expression for genes in these different classes (Figure 2B). This analysis revealed a clear association between expression pleiotropy (i.e., fraction of total samples in which genes are expressed) and the severity of phenotypes. Essential genes that are not associated with disease are likely enriched for embryonic lethality and are, congruently, the most pleiotropic. The group of genes that when mutated range from lethality to causing disease (often developmental disorders affecting multiple organs) are less pleiotropic than embryonic lethals but are more pleiotropic than genes only associated with disease (both p values = 2×10^{-16}, Wilcoxon rank sum test, two sided; Figure 2B). Finally, nonlethal disease genes are more pleiotropic than genes unassociated with any deleterious phenotypes (p value = 2×10^{-5}; Figure 2B). A similar association is obtained when looking independently at organ and time specificity (Figure S2A). The breadth of developmental expression is therefore positively correlated with phenotypic severity.

Human diseases differ in terms of severity, age of onset, and organs affected, all of which should be reflected in the spatiotemporal expression profiles of the underlying disease genes. Therefore, we looked at the time and organ specificity of genes associated with different classes of disease (Stenson et al., 2017) (Figure 2C). As expected, the specificity of the spatiotemporal profiles of disease genes differs considerably among disease classes. Genes implicated in developmental disorders, cancer, and diseases of the nervous system tend to be ubiquitously expressed, whereas genes causing heart and reproductive diseases tend to have more restricted expression (Figure 2C).

Further insights were obtained by analyzing the temporal trajectories of disease genes within the organs they affect. To do this, we used a soft clustering approach to identify the most common expression profiles in each organ and assigned each gene a probability of belonging to each of the clusters (STAR Methods; Table S2). Disease genes are enriched within specific clusters, which are disease and organ specific. For example, genes associated with heart disease are significantly enriched among genes characterized by a progressive increase in expression throughout heart development (Figure S2B; Bonferroni-corrected p value = 2×10^{-6}, binomial test), whereas genes associated with metabolic diseases are enriched among genes that exhibit a strong upregulation in the liver in the first months after birth (Figure S2C; Bonferroni-corrected p value = 3×10^{-15}, binomial test). Within the brain, we focused on the temporal trajectories of genes associated with three neurodevelopmental disorders: primary microcephaly, autism spectrum disorders, and schizophrenia (STAR Methods). Consistent with these disorders having different etiologies and ages of onset, the associated genes are significantly enriched among distinct temporal profiles in the brain (Figure 2D). Genes causing primary microcephaly show their highest expression at the earliest developmental

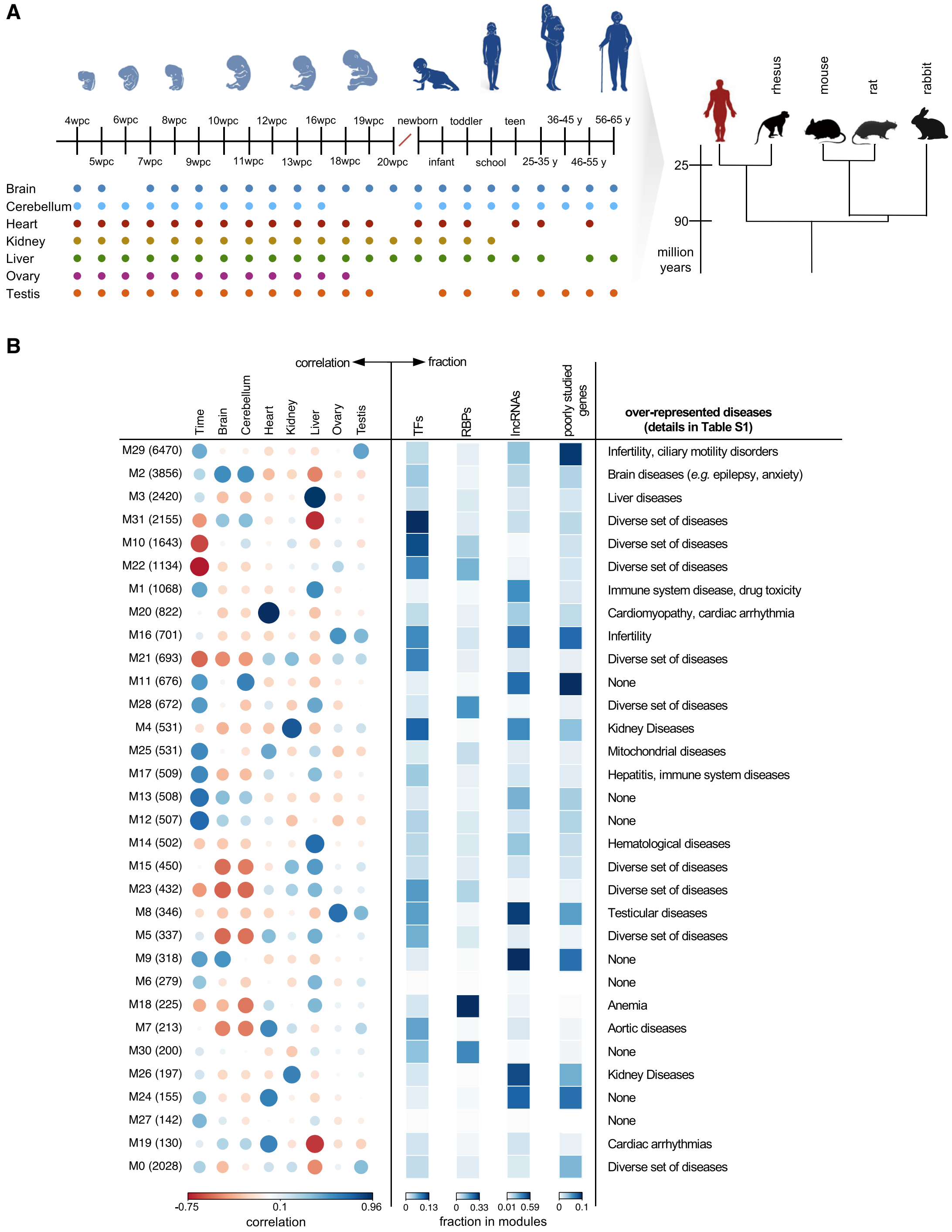

Figure 1. An Expression Atlas of Human Organ Development

(A) Description of the dataset. The dots mark the developmental stages sampled in each organ (median of two replicates), and its colors reflect the colors used throughout the figures to represent each organ.

(B) Modules in the gene co-expression network (number of genes in each module in parentheses). The left panel shows the correlations between each module and the expression levels in each organ or developmental time (full developmental profiles in Figure S1A). The color intensity and the size of the circles are proportional to the correlation coefficients. A positive correlation with developmental time (first column) means higher expression late in development, and a negative correlation means higher expression early in development. The middle panel shows the fraction of genes in each module that correspond to TFs, RBPs, developmentally dynamic lncRNAs, and poorly studied protein-coding genes (i.e., genes with three or fewer publications). The right panel shows examples of overrepresented diseases (FDR <1%, hypergeometric test). Table S1 lists the top five biological and disease enrichments (FDR <1%) for each of the 32 modules. The modules are ordered vertically by decreasing number of genes. Module 0 (bottom) includes genes not assigned to any of the other modules.

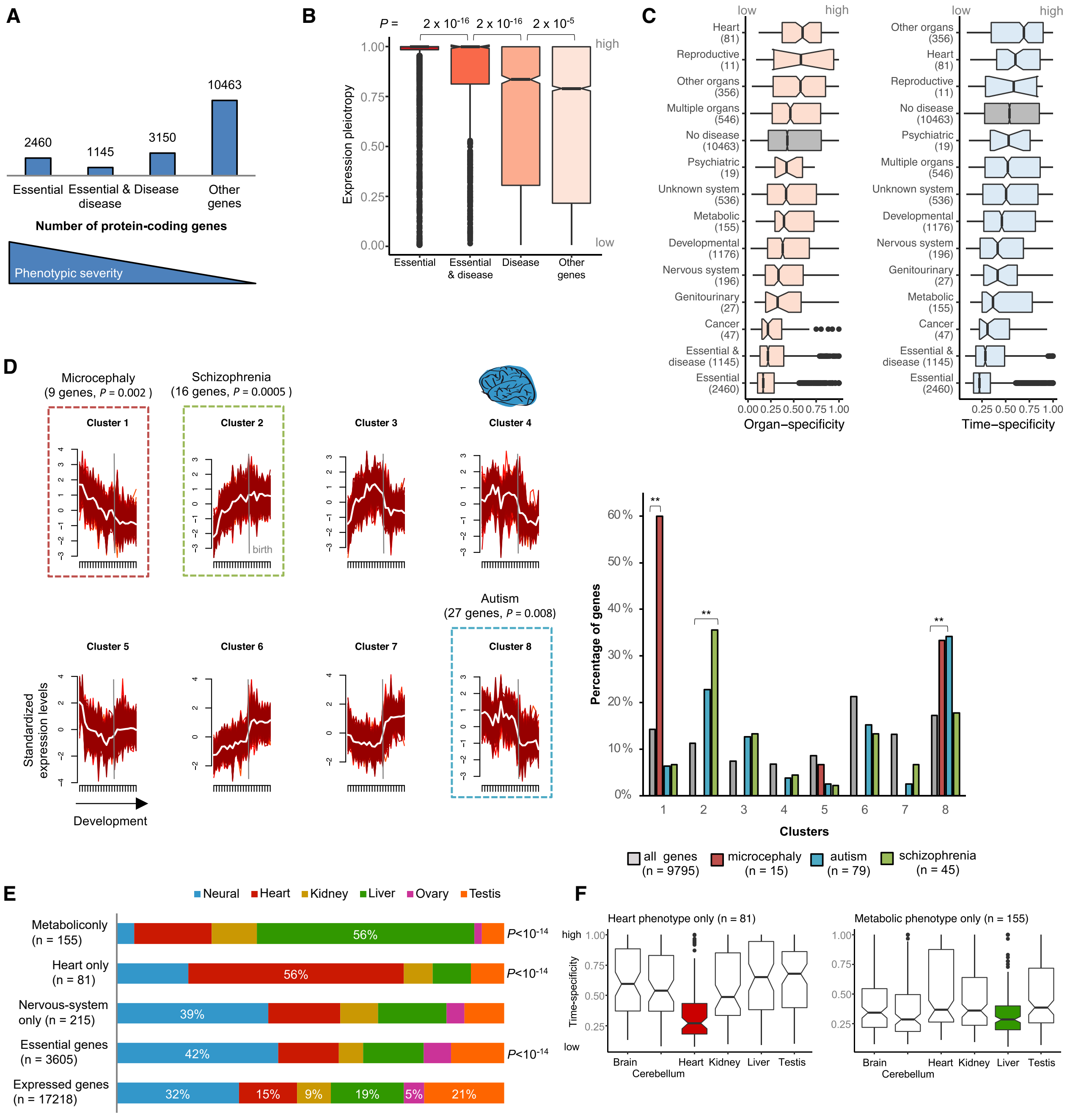

Figure 2. Spatiotemporal Profiles of Disease Genes

(A) Number of expressed (RPKM >1) protein-coding genes in different classes of phenotypic severity.

(B) Expression pleiotropy of genes in different classes of phenotypic severity (p values from Wilcoxon rank sum test, two sided; the number of genes in each class is shown in A).

(C) Organ and time specificity (median across organs) of genes associated with different disease classes (number of genes in each class in parentheses). Genes are only assigned to one class; those affecting multiple organs appear in the classes "Multiple organs," "Developmental", or "Other organs" depending on whether they affect multiple organs that include at least one of the organs in this study, are associated with developmental phenotypes, or affect organs that are not part of this study, respectively.

(D) Genes associated with primary microcephaly, autism, and schizophrenia are significantly enriched in distinct expression clusters in the brain (binomial tests with Bonferroni correction). On the left are the developmental profiles for the clusters identified through soft clustering of the brain developmental samples. The y axis shows standardized expression levels (STAR Methods). The profile of each gene in the cluster is shown in red; the white line shows the cluster center. The genes associated with each disorder are significantly enriched in only one of the eight clusters (right).

(legend continued on next page)

stages followed by a progressive decrease in expression (Figure 2D; 9 out of 15 genes, Bonferroni-corrected p value = 0.002, binomial test), whereas genes implicated in schizophrenia show the opposite profile: a progressive increase in expression throughout development (16 out of 45 genes, Bonferroni-corrected p value = 0.0005). Genes associated with autism are expressed throughout prenatal development and subsequently display a sharp decrease in expression near birth (Figure 2D; 27 out of 79 genes, Bonferroni-corrected p value = 0.008, consistent with Satterstrom et al., 2020). The two temporal profiles enriched with microcephaly- and autism-associated genes are also enriched with essential genes (Bonferroni-corrected p value $< 10^{-15}$, binomial test).

Organ-Specific Phenotypes of Ubiquitously Expressed Genes

Most disease genes that we analyzed are associated with phenotypes in multiple organs (3,060 genes [71%]), but this still leaves hundreds of genes that affect exclusively one organ. Many of these genes with organ-specific phenotypes present a puzzle in biomedical research, because their expression is not organ specific (Barshir et al., 2018; Lage et al., 2008). Our analysis of developmental transcriptomes further highlights this issue. Genes associated with organ-specific phenotypes exhibit dynamic temporal profiles in a similar number of organs as genes associated with phenotypes across multiple organs (i.e., median of four organs for both gene sets; Figure S2D). This raises the intriguing question of how mutations that predominantly disrupt the coding sequences of genes employed during the development of multiple organs result in diseases that are organ specific.

While a number of factors may explain this phenomenon, including alternative splicing (Omer Javed et al., 2018), functional redundancy (Barshir et al., 2018), and dependency on the characteristics of specific cell types like protein-misfolding diseases in long-lived neurons, it has been suggested that pathologies tend to be associated with the organ where the genes display elevated expression (Lage et al., 2008). This prompted us to ask if genes associated with organ-specific diseases exhibit their maximum expression during the development of the affected organ. We focused on heart, neurodevelopmental, psychiatric, and metabolic diseases (the latter tested in association with the liver) and found a strong association between the organ of maximum expression during development and the organ where the pathology manifests (Figure 2E). We found that 56% of the genes exclusively associated with heart disease show maximal expression in the heart (versus 15% for all genes, Bonferroni-corrected p value = 9×10^{-15}, binomial test; Figure 2E), 56% of the genes with an exclusively metabolic phenotype show maximal expression in the liver (versus 19% for all genes, Bonferroni-corrected p value = 9×10^{-15}; Figure 2E), and 39% of the genes exclusively associated with neurodevelopmental diseases show maximal expression in the brain (versus 32% for all genes, Bonferroni-corrected p value = 0.1; Figure 2E).

At least for heart disease, the duration of gene expression may also help explain organ-specific pathologies. Genes expressed in multiple organs that have heart-specific phenotypes are ubiquitously expressed during heart development but show a significantly higher time specificity (i.e., shorter expression window) in the other organs (all Bonferroni-corrected p values $< 10^{-4}$, Wilcoxon rank sum test, two sided; Figure 2F). In contrast, the duration of gene expression does not appear to underlie metabolic- or neurodevelopmental-specific phenotypes, as we see no difference in the time specificity of genes in the affected organs versus the others (Figures 2F and S2E). Overall, the association of pathology with the level of gene expression and, to a lesser extent, the duration of gene expression suggests that the development of organ-specific pathologies can at least in some cases be explained by differences in the abundance of the cell types that express the mutated gene in the different organs.

Most Disease Genes Have Orthologs in Mammalian Models

The extensive use of mice, rats, and other mammals in biomedical research is predicated on the assumption of an overall conservation of developmental programs between humans and these species. This assumption has been largely supported by comparative analyses of developmental expression profiles (Cardoso-Moreira et al., 2019) and comparative analyses of the human and mouse *trans*-acting regulatory circuitry (Stergachis et al., 2014). However, there are exceptions to this overall conservation that can profoundly impact the translatability of phenotypes between humans and other species.

One exception applies to genes that have duplicated recently in human history and therefore do not have a strict 1:1 orthology relationship with other species. The lack of 1:1 orthologs poses challenges to the study of recently evolved human genes, which is reflected in younger genes (i.e., more recently originated) being more poorly studied than older genes (as measured by the number of publications; Figure S3; see also Zhang et al., 2012). In this context, it is notable that the younger genes are, the less likely they are to be associated with disease (with the caveat that they are also more poorly studied) (Figure 3A). While 29% of human genes with 1:1 orthologs across vertebrates are associated with disease, the same is true for only 1% of human-specific genes (Figure 3A). One likely explanation is that the younger genes are, the more organ- and time-specific they are also likely to be (Figure 3B; see also Milinkovitch et al., 2009). This relation is important because (as shown above) the more specifically genes are expressed during development (temporally and spatially), the less severe are the phenotypes associated with mutations in those genes (Figure 2B).

Of the 4,295 disease-associated genes that are expressed in the human developmental atlas, only 155 (4%) do not have a 1:1 ortholog in at least one of four mammals commonly used to study human physiology: mouse, rat, rabbit, and rhesus macaque. Of these 155 genes, most (85%) originated before

(E) Organs where genes associated with organ-specific phenotypes show maximum expression. p values are from binomial tests after Bonferroni correction.

(F) Time specificity in the different organs of genes with heart- and metabolic-specific phenotypes.

In (B), (C), and (F), the boxplots depict the median ±25th and 75th percentiles, with the whiskers at 1.5 times the interquartile range.

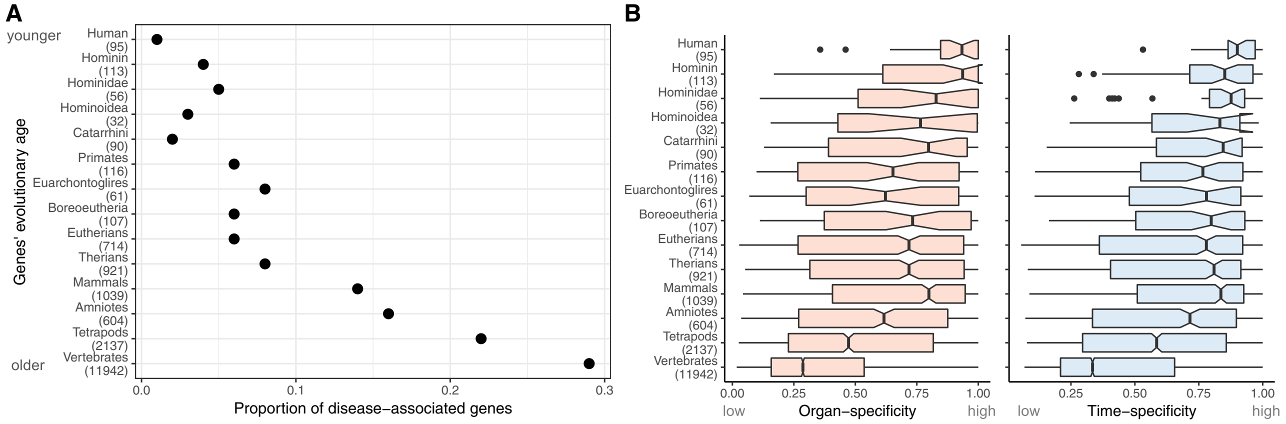

Figure 3. Recently Originated Genes Are Infrequently Associated with Disease

(A) The proportion of disease-associated genes decreases for groups of genes of successively younger evolutionary ages. 2,231 genes do not have an age assignment, and of these, 310 (13%) are associated with human disease.

(B) Organ and time specificity of human genes with different evolutionary ages.

In (A) and (B), the youngest genes are those that are human specific (top), and the oldest are those shared across vertebrates (bottom). In parentheses are the number of genes in each age class.

primates split from the glires lineage (i.e., rodents and rabbits), which indicates gene loss events in the non-human lineages or genome annotation problems (Shao et al., 2019). Overall, these analyses suggest that most human disease genes could in principle be studied in one of the four mammalian models.

Presence/Absence Expression Differences Are Rare between Species

We next evaluated the extent of differences between human and each of the four model species in terms of stark differences in spatiotemporal profiles of 1:1 orthologs: presence/absence of gene expression in a given organ or large differences in expression pleiotropy across multiple organs. Our analyses showed that differences between human and the other species in terms of presence/absence of gene expression in an organ are rare. In a comparison between human and mouse, only 1%–3% of protein-coding genes (177–372 genes depending on the organ) are robustly expressed (reads per kilobase of exon per million mapped reads [RPKM] $\geq$ 5) in human, but not in mouse (RPKM $\leq$ 1). These percentages are similar for the comparisons with the other species (i.e., 1%–2% of genes robustly expressed in human are not expressed in rat, rabbit, or rhesus macaque). Although rare, these differences include disease genes. For example, among genes robustly expressed in heart in human, but not in mouse, are 17 genes associated with heart disease (similar to the expected number given presence/absence differences in the heart). These include *NKX2-6*, which causes conotruncal heart malformations in human (Ta-Shma et al., 2014) that, congruently, are not recapitulated by a mouse knockout (Bello et al., 2015). The developmental profile of *NKX2-6* in the human heart is ancestral; heart expression was lost specifically in rodents, and this is therefore an example of a disease gene that would be better studied in the rabbit (Figure 4A). Genes associated with neurological diseases are depleted among the set of genes expressed in the human, but not in the mouse, brain (11 differ versus 28 expected, p value = 4×10^{-4}, binomial test). Among the exceptions is *CHRNA2*, a gene expressed in the human brain starting at birth that has been implicated in epilepsy (Aridon et al., 2006; Conti et al., 2015). Once again, and congruently, this clinical phenotype is not recapitulated in the mouse knockout (Bello et al., 2015) (Figure 4B).

The breadth of spatiotemporal expression is also very similar between human genes and their orthologs in mouse, rat, rabbit, and rhesus macaque. They are highly correlated in terms of their organ specificity (Pearson's r = 0.86, all Bonferroni-corrected p values < 10^{-15}), time specificity (r = 0.67–0.84 for individual organs and 0.83–0.84 for median time specificity, all Bonferroni-corrected p values < 10^{-15}), and, therefore, global expression pleiotropy (r = 0.85–0.88, all Bonferroni-corrected p values < 10^{-15}). There are only 141 genes expressed in at least half the human samples but in fewer than 10% of the mouse samples, and 172 genes with the opposite pattern (Figure S4). These genes are depleted for essential genes (4% versus 11% in entire dataset, p value = 8×10^{-6}, binomial test) and disease genes (16% versus 26% in entire dataset, p value = 0.02, binomial test). Similar results are obtained in comparisons between human and each of the other species (Figure S4). Together with the results above, these analyses indicate that differences in the breadth and presence/absence of gene expression between humans and other species are confined to a small set of genes. However, when present, they can translate into relevant phenotypic differences that are relevant to biomedical research.

Organ Developmental Trajectory Differences Are Common

Although stark differences in gene expression are rare between humans and other species, we previously showed that it is not uncommon for genes with broad spatiotemporal profiles to evolve new organ-specific developmental trajectories (Cardoso-Moreira et al., 2019). In that work, we studied the evolution

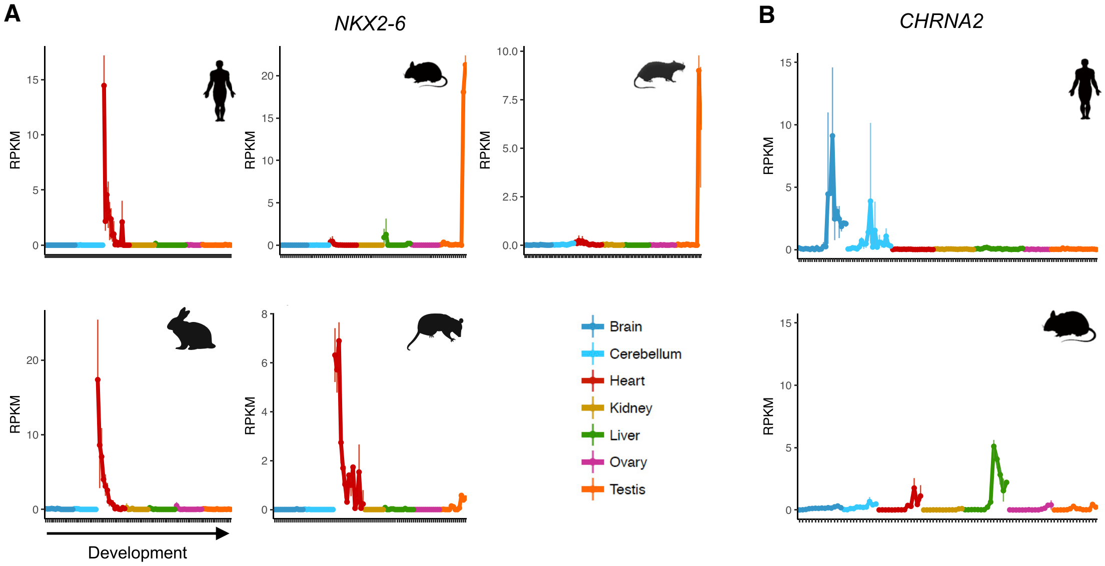

Figure 4. Suitability of the Mouse as a Model

(A) Developmental profile of *NKX2-6* in human, mouse, rat, rabbit, and opossum (marsupial). *NKX2-6* is robustly expressed in the human heart, but not in mouse, and the conotruncal heart malformations observed in human are not recapitulated by a mouse knockout. The human heart profile of *NKX2-6* is ancestral, as it is similar to the profiles in rabbit and opossum.

(B) Developmental profile of *CHRNA2* in human and mouse. *CHRNA2* is robustly expressed in the human brain, but not in mouse, and the epileptic phenotypes observed in human are not recapitulated by a mouse knockout.

In (A) and (B), the x axis shows samples for each organ ordered from early to late development (stages sampled in Table S4), and the y axis shows expression levels in reads per kilobase of exon model per million mapped reads (RPKM).

of developmental expression programs across distantly related mammals using a phylogenetic approach that assigned changes in organ temporal trajectories to individual phylogenetic branches (Cardoso-Moreira et al., 2019). This limited the number of human genes that could be tested for trajectory changes (1,871–3,980 genes depending on the organ), because jointly analyzing distantly related species considerably reduced the number of available 1:1 orthologs, and trajectory changes had to be unambiguously assigned to one branch of the phylogenetic tree.

Here, we aimed to identify differences in organ developmental trajectories between human and each of the four mammalian models for the maximum number of human genes. Therefore, we compared the developmental profiles of human genes with their orthologs in each of the species separately, in a pairwise manner. Doing pairwise comparisons allowed us to double or triple (depending on the organ) the number of human genes that could be evaluated for organ trajectory differences (e.g., 5,253–8,666 genes in human-mouse comparisons). We used a two-step approach. First, we used soft clustering to identify the main types (or clusters) of temporal trajectories in each organ jointly for human and non-human orthologs (STAR Methods). Second, we identified all instances where the human gene and its ortholog were assigned to different clusters (5% false discovery rate [FDR]; STAR Methods; Figures 5 and S5). We were interested in genes that differ between species in the entirety of their temporal trajectory (e.g., genes assigned to cluster 0 in one species and to cluster 1 in another in Figure 5 for the brain) and in genes that differ in only part of the time series (e.g., genes assigned to cluster 2 in one species and to cluster 6 in the other in Figure 5 for the brain).

Overall, we identified thousands of genes with different developmental trajectories between human and each of the other species (Figure 6A; Table S3). Because we performed our trajectory comparisons in a pairwise fashion, we could examine our calls across trios of species (e.g., mouse, rat, and human) to evaluate the sensitivity and specificity of our approach. Genes inferred to have a similar trajectory between mouse and rat and between mouse and human should also have a similar trajectory between human and rat. This was true for ~96% of the calls, consistent with our 5% FDR threshold (Figure S6A). Conversely, genes inferred to be similar between mouse and rat and different between mouse and human should have a different trajectory between human and rat. This was true for 65%–82% of the calls, suggesting our approach is conservative when calling for species differences (Figure S6A). We further evaluated the consistency of our trajectory calls using an external dataset generated by the PsychENCODE consortium that compared gene expression profiles between human and rhesus macaque for 11 different areas of the neocortex for the prenatal, postnatal and adult periods (Li et al., 2018; Zhu et al., 2018). In support of our approach, the genes that we identified as having different brain developmental trajectories between human and rhesus macaque were also significantly more likely to show spatial and temporal differences between these two species in the PsychENCODE dataset (Figure S6B; p value = 1×10^{-5}, Wilcoxon rank sum test).

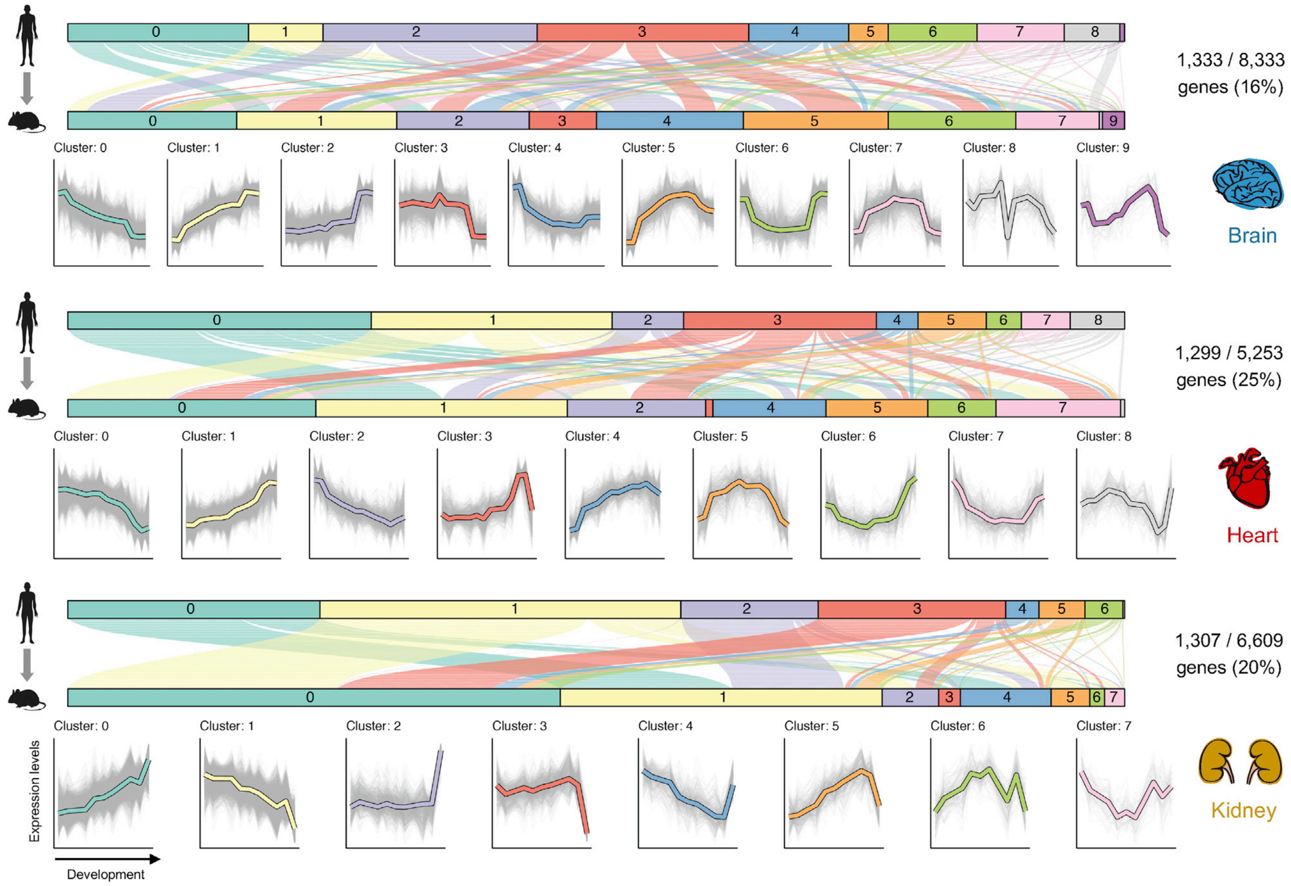

Figure 5. Developmental Trajectory Differences between Human and Mouse in Brain, Heart, and Kidney
We used soft clustering to group human genes and their mouse orthologs according to their temporal expression in each organ. The genes for which the human and mouse orthologs were assigned to a different cluster are shown on top. Each line represents a pair of human-mouse orthologs with a significant trajectory difference and shows the cluster assignment in human (top) and mouse (bottom). The lines are colored according to the human cluster assignment. The clusters identified in each organ are shown below (gray lines correspond to individual genes and the colored lines to the cluster center). The y axis shows the log normalized expression levels, and the x axis shows the samples ordered from early to late development. The same analysis for cerebellum, kidney, and testis is shown in Figure S5.

As expected, a smaller fraction of genes differ between human and rhesus macaque (diverged ~29 million years ago) than between human and mouse, rat, or rabbit (diverged ~90 million years ago) (Figure 6A). However, for all organs and despite the same divergence time, a higher proportion of genes differ between human and mouse than between human and rabbit (Figure 6A). In human-mouse comparisons, 51% of the genes tested show a different developmental trajectory in at least one of the organs (6,816 out of 13,471 genes tested). In human-rat comparisons, this percentage is 45% (5,459 out of 12,155 genes), and in human-rabbit comparisons, it is only 38% (4,568 out of 11,731 genes). These species differences are robust to using the same gene set of 1:1 orthologs for all pairwise species comparisons (Figure S6C) and using different clustering parameters (Figure S6D). The observation that there are more genes with trajectory differences between human and mouse than between human and rabbit is consistent with the rodent lineage having evolved a larger number of trajectory differences (Cardoso-Moreira et al., 2019) and suggests that rabbits have some advantages over mice for studying human biology.

Next, we set to characterize the genes with trajectory differences between humans and the other species. Below, we report on the human-mouse comparison, but the results are consistent across all species comparisons. An analysis of the GTEx dataset (Lonsdale et al., 2013), which contains human gene expression profiles for hundreds of adults across multiple tissues, shows that genes with trajectory differences between species do not show greater variation in gene expression among humans (Figure S6E; STAR Methods). Therefore, the species differences are not a consequence of the genes involved having more variable expression profiles. At the level of the coding sequence, we found that genes with trajectory differences are under similar levels of functional constraint as genes with similar trajectories. For most organs, genes in both groups show similar levels of intolerance to loss-of-function mutations (Figure S7A). The exception are the neural tissues (brain and cerebellum), where genes with trajectory differences show more tolerance to

CellPress
OPEN ACCESS

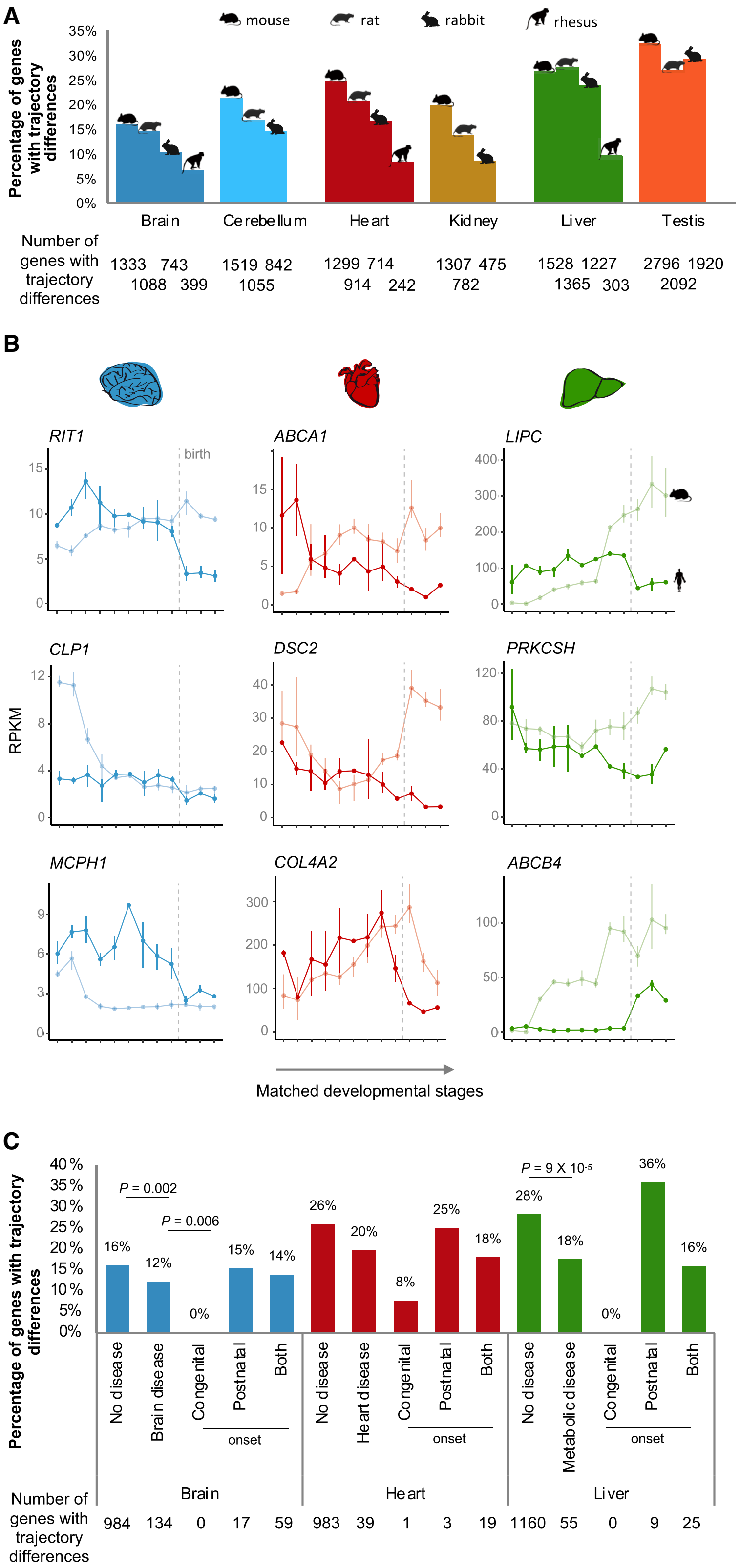

Figure 6. Developmental Trajectory Differences

(A) Percentage of genes in each organ that have different trajectories between human and mouse, rat, rabbit, and rhesus macaque (because of the shorter time series, this analysis was only performed for brain, heart, and liver in rhesus). The sets of genes compared differ between species as they correspond to the total number of 1:1 orthologs available between human and each species. Figure S6C shows the same analysis but using the same set of 1:1 orthologs across all species (similar results). Table S3 lists the genes tested for trajectory differences and those found to be significantly different between human and each of the other species.

(B) Examples of human disease genes with different developmental trajectories between human and mouse in the affected organ (FDR <5%).

(C) Percentage of genes in brain, heart, and liver that differ in trajectories between human and mouse. Bonferroni-corrected p values for comparisons between disease and non-disease genes are from Fisher's exact tests, and Bonferroni-corrected p values for comparisons of disease genes with different ages of onset are from binomial tests.

functional mutations than those with similar trajectories (Figure S7A). Similar relationships apply to genes' intolerance to copy-number variation (duplications and deletions); genes with trajectory differences in the neural tissues are slightly more tolerant to copy-number variation than genes with similar trajectories, and no differences are observed for the other organs (Figure S7B). Interestingly, genes with different trajectories in the brain (but not other organs) are also enriched among a set of genes identified as carrying signs of positive selection in their coding sequences across mammals (Kosiol et al., 2008) (Benjamini-Hochberg corrected p value = 0.02, Fisher's exact test). Finally, across organs, genes with trajectory differences are enriched for protein metabolism (Benjamini-Hochberg corrected p value = 0.0001, hypergeometric test).

Organ Trajectory Differences among Disease Genes

The genes depicted in Figure 6B are associated with diseases that affect the organ in which human and mouse display different trajectories. For these genes, the disease etiology may not be fully recapitulated by mouse models. The mouse knockouts are still expected to affect the development of the organ associated with the disease, but the cellular and developmental context of the phenotypes in mouse could differ substantially from those in human. It is therefore noteworthy that genes associated with human disease are less likely than non-disease genes to differ in their trajectories between human and mouse (Figure 6C). Genes causing diseases that affect the brain and liver are depleted for trajectory differences between human and mouse in each of the organs (Figure 6C; p value = 0.002 for the brain, p value = 0.1 for the heart, and p value = 9×10^{-5} for the liver, Fisher's exact test after Bonferroni correction). This is also true for comparisons between human and other species (Figure S7C). Nevertheless, that still leaves more than 200 disease genes whose developmental profiles may not be fully recapitulated in the mouse (Figure 6C; ~40% fewer genes in the rabbit; Figure S7C).

We further asked if genes underlying diseases with different ages of onset are equally likely to differ in their organ trajectories between human and mouse. Although the number of disease genes associated with an exclusive congenital or exclusive postnatal onset is low, we found that genes with congenital onsets rarely differ in terms of their developmental trajectories between human and mouse (i.e., only 1 out of 82 genes causing disease in the brain, heart, or liver; Figure 6C), whereas genes with postnatal onsets are more likely to show differences (Figure 6C; same applies to comparisons between human and other species; Figure S7C). This suggests that diseases with a congenital onset may be easier to study in model species than diseases whose phenotypic manifestations start later in life.

DISCUSSION

In order to shed new light on the causes and phenotypic manifestations of human diseases, we integrated a resource of human organ developmental gene expression profiles with datasets of human essential and disease genes. We found that the breadth of developmental expression is positively correlated with phenotypic severity and that it varies considerably among disease classes. Disease-associated genes are enriched within specific developmental modules in the organs affected. For example, genes associated with different brain developmental disorders show distinct temporal profiles during brain development. Overall, we found a clear association between spatiotemporal profiles and the phenotypic manifestations of diseases.

The analysis of developmental transcriptomes further strengthened the apparent paradox of ubiquitously expressed genes often having organ-specific phenotypes (Barshir et al., 2018; Hekselman and Yeger-Lotem, 2020; Lage et al., 2008). We could not distinguish genes associated with organ-specific phenotypes from those associated with multi-organ phenotypes based on the breadth of spatiotemporal profiles, which were similar. However, for genes associated with organ-specific phenotypes, we found a strong association between the organ affected and the organ of maximal expression during development. This association suggests that some organ-specific pathologies could be explained by differences between organs in the spatial and temporal abundance of the cells expressing the mutated gene.

Gene expression links genes with their organismal phenotypes and hence offers a direct means to compare both across species. This is not without its challenges. Gene expression differences can relate to phenotypes in complex ways, genes that have duplicated cannot be directly compared across species, and cross-species comparisons of whole organs cannot directly address the extent to which differences in cell abundances underly changes in gene expression (discussed below) (Pantalacci and Semon, 2014). Despite these challenges, comparing gene expression between species for matching organs and developmental stages provides a powerful tool to evaluate the likelihood that insights obtained from studies in model species can be directly transferable to human. Within this context, it is notable that most (96%) genes associated with human disease have 1:1 orthologs in commonly used mammalian model species and can, therefore, be directly compared.

Overall, we found that stark changes in gene expression (e.g., presence/absence of expression) are rare between species. However, instances of such changes sometimes occur in disease genes, and in these cases, the differences that we identify may explain why animal models fail to recapitulate human phenotypes. In contrast, we found that differences in temporal trajectories during organ development are common between humans and other species. Approximately half of human genes exhibit a different developmental trajectory from their mouse orthologs in at least one of the organs. In further support of the use of model organisms for disease research, we found that disease genes are less likely to differ than the other genes. Nevertheless, we still identified more than 200 genes known to be causally associated with brain, heart, and/or liver disease that differ in their developmental trajectories between human and mouse in the affected organ. It is unclear how the subtler differences in developmental trajectories that we have identified (e.g., *COL4A2* in Figure 6B) translate at the level of phenotypes. Sill, we suggest that for disease genes with different temporal trajectories between human and mouse, the existing mouse models of human diseases should undergo extra scrutiny, and the possibility of studying alternative models should be carefully considered.

When human disease genes with organ trajectory differences are studied in animal models, their genetic manipulation (e.g., knockout) is still expected to affect the functioning of the organ affected by the human disease. Genes with trajectory differences show dynamic temporal profiles in both species, suggesting the orthologs play roles during organ development in the two species, but potentially different ones. This poses considerable challenges for phenotyping efforts of animal models of human disease, with abnormal organ function expected in the model species when genes have both similar and different developmental trajectories. Luckily, efforts to systematically and comprehensively phenotype animal models of human disease are currently underway that will address these challenges (Cacheiro et al., 2019; Meehan et al., 2017).

How differences in organ developmental trajectories translate into phenotypic differences between species will depend to a large extent on the reasons for the trajectory differences. Trajectory differences can be created by gene expression differences between species in homologous cell types, differences between species in cellular composition, and/or differences between species in the cell types that express orthologous genes. All of these non-mutually exclusive possibilities can decrease the likelihood that the phenotype associated with a human gene will be fully recapitulated in a model species. However, the magnitude of the phenotypic differences is expected to differ depending on the underlying reasons. For example, trajectory differences created by changes in the identity of the cell types that express an orthologous gene could lead to the greatest phenotypic divergence. Such differences would be highly relevant to interpreting animal models of human disease genes with adult onsets, because they would suggest a distinct cellular basis for the disease in humans and in the model species. Endeavors that seek to clarify the causes of trajectory differences therefore represent a key next step, and the application of single-cell technologies across species will greatly aid these efforts (Bakken et al., 2020; Shami et al., 2020; Xue et al., 2013).

Gene expression is only one of several steps connecting genes to their phenotypes (Buccitelli and Selbach, 2020). Similarities and differences in gene expression between species will not always translate into conserved and divergent phenotypes, respectively. This notwithstanding, detailed comparisons of developmental gene expression profiles, as performed here, can substantially help to assess the translatability of the knowledge gathered for individual genes from model species to humans.

STAR★METHODS

Detailed methods are provided in the online version of this paper and include the following:

- KEY RESOURCES TABLE
- RESOURCE AVAILABILITY
  - Lead contact
  - Materials availability
  - Data and code availability
- METHOD DETAILS
  - Resource
  - Gene co-expression networks
  - Inherited disease genes
  - Age of human genes and orthology
  - Organ developmental trajectories
  - Characterization trajectory differences
- QUANTIFICATION AND STATISTICAL ANALYSIS

SUPPLEMENTAL INFORMATION

Supplemental Information can be found online at https://doi.org/10.1016/j.celrep.2020.108308.

ACKNOWLEDGMENTS

We thank S. Anders, R. Arguello, M. Sanchez Delgado, M. Sepp, T. Studer, Y.E. Zhang, and members of the Kaessmann group for discussions. D.N.C. and M.M. are in receipt of financial support from Qiagen through a License Agreement with Cardiff University. This research was supported by grants from the European Research Council (615253, OntoTransEvol) and Swiss National Science Foundation (146474) to H.K. and Marie Curie FP7-PEOPLE-2012-IIF (329902) to M.C.-M.

AUTHOR CONTRIBUTIONS

M.C.-M. and H.K. conceived the study. M.C.-M. performed most analyses, with contributions from I.S. B.V. and W.H. contributed to the analyses on trajectory differences. M.M. and D.N.C. contributed to the analyses on human inherited disease. M.C.-M. wrote the manuscript, with input from all authors.

DECLARATION OF INTERESTS

The authors declare no competing interests.

Received: July 4, 2019
Revised: August 16, 2020
Accepted: October 5, 2020
Published: October 27, 2020

REFERENCES

Anders, S., and Huber, W. (2010). Differential expression analysis for sequence count data. Genome Biol. *11*, R106.

Aridon, P., Marini, C., Di Resta, C., Brilli, E., De Fusco, M., Politi, F., Parrini, E., Manfredi, I., Pisano, T., Pruna, D., et al. (2006). Increased sensitivity of the neuronal nicotinic receptor α2 subunit causes familial epilepsy with nocturnal wandering and ictal fear. Am. J. Hum. Genet. *79*, 342–350.

Auguie, B. (2016). gridExtra: miscellaneous functions for “grid” graphics. R package version 2.2. 1.

Bakken, T.E., Miller, J.A., Ding, S.L., Sunkin, S.M., Smith, K.A., Ng, L., Szafer, A., Dalley, R.A., Royall, J.J., Lemon, T., et al. (2016). A comprehensive transcriptional map of primate brain development. Nature *535*, 367–375.

Bakken, T.E., Jorstad, N.L., Hu, Q., Lake, B.B., Tian, W., Kalmbach, B.E., Crow, M., Hodge, R.D., Krienen, F.M., Sorensen, S.A., et al. (2020). Evolution of cellular diversity in primary motor cortex of human, marmoset monkey, and mouse. bioRxiv.

Barshir, R., Hekselman, I., Shemesh, N., Sharon, M., Novack, L., and Yeger-Lotem, E. (2018). Role of duplicate genes in determining the tissue-selectivity of hereditary diseases. PLoS Genet. *14*, e1007327.

Bartha, I., di Iulio, J., Venter, J.C., and Telenti, A. (2018). Human gene essentiality. Nat. Rev. Genet. *19*, 51–62.

Bello, S.M., Smith, C.L., and Eppig, J.T. (2015). Allele, phenotype and disease data at Mouse Genome Informatics: improving access and analysis. Mamm. Genome *26*, 285–294.

Benjamini, Y., and Hochberg, Y. (1995). Controlling the false discovery rate: a practical and powerful approach to multiple testing. J. R. Stat. Soc. B *57*, 289–300.

Bruneau, B.G. (2013). Signaling and transcriptional networks in heart development and regeneration. Cold Spring Harb. Perspect. Biol. *5*, a008292.

Buccitelli, C., and Selbach, M. (2020). mRNAs, proteins and the emerging principles of gene expression control. Nat. Rev. Genet. *21*, 630–644.

Cacheiro, P., Haendel, M.A., Smedley, D., Meehan, T., Mason, J., Mashhadi, H.H., Muñoz-Fuentes, V., Tocchini, G., Lloyd, K.K.C., McKerlie, C., et al.; International Mouse Phenotyping Consortium and the Monarch Initiative (2019). New models for human disease from the International Mouse Phenotyping Consortium. Mamm. Genome *30*, 143–150.

Cardoso-Moreira, M., Halbert, J., Valloton, D., Velten, B., Chen, C., Shao, Y., Liechti, A., Ascenção, K., Rummel, C., Ovchinnikova, S., et al. (2019). Gene expression across mammalian organ development. Nature *571*, 505–509.

Conti, V., Aracri, P., Chiti, L., Brusco, S., Mari, F., Marini, C., Albanese, M., Marchi, A., Liguori, C., Placidi, F., et al. (2015). Nocturnal frontal lobe epilepsy with paroxysmal arousals due to CHRNA2 loss of function. Neurology *84*, 1520–1528.

DeFalco, T., and Capel, B. (2009). Gonad morphogenesis in vertebrates: divergent means to a convergent end. Annu. Rev. Cell Dev. Biol. *25*, 457–482.

Dickinson, M.E., Flenniken, A.M., Ji, X., Teboul, L., Wong, M.D., White, J.K., Meehan, T.F., Weninger, W.J., Westerberg, H., Adissu, H., et al.; International Mouse Phenotyping Consortium; Jackson Laboratory; Infrastructure Nationale PHENOMIN, Institut Clinique de la Souris (ICS); Charles River Laboratories; MRC Harwell; Toronto Centre for Phenogenomics; Wellcome Trust Sanger Institute; RIKEN BioResource Center (2016). High-throughput discovery of novel developmental phenotypes. Nature *537*, 508–514.

Finucane, H.K., Reshef, Y.A., Anttila, V., Slowikowski, K., Gusev, A., Byrnes, A., Gazal, S., Loh, P.R., Lareau, C., Shoresh, N., et al.; Brainstorm Consortium (2018). Heritability enrichment of specifically expressed genes identifies disease-relevant tissues and cell types. Nat. Genet. *50*, 621–629.

Futschik, M.E., and Carlisle, B. (2005). Noise-robust soft clustering of gene expression time-course data. J. Bioinform. Comput. Biol. *3*, 965–988.

Gerrelli, D., Lisgo, S., Copp, A.J., and Lindsay, S. (2015). Enabling research with human embryonic and fetal tissue resources. Development *142*, 3073–3076.

Gerstberger, S., Hafner, M., and Tuschl, T. (2014). A census of human RNA-binding proteins. Nat. Rev. Genet. *15*, 829–845.

Giudice, J., Xia, Z., Wang, E.T., Scavuzzo, M.A., Ward, A.J., Kalsotra, A., Wang, W., Wehrens, X.H.T., Burge, C.B., Li, W., and Cooper, T.A. (2014). Alternative splicing regulates vesicular trafficking genes in cardiomyocytes during postnatal heart development. Nat. Commun. *5*, 3603.

Hekselman, I., and Yeger-Lotem, E. (2020). Mechanisms of tissue and cell-type specificity in heritable traits and diseases. Nat. Rev. Genet. *21*, 137–150.

Hensman, J., Rattray, M., and Lawrence, N.D. (2012). Fast variational inference in the conjugate exponential family. In Advances in Neural Information Processing Systems (NIPS 2102), pp. 2888–2896.

Hensman, J., Lawrence, N.D., and Rattray, M. (2013). Hierarchical Bayesian modelling of gene expression time series across irregularly sampled replicates and clusters. BMC Bioinformatics *14*, 252.

Hensman, J., Rattray, M., and Lawrence, N.D. (2015). Fast nonparametric clustering of structured time-series. IEEE Trans. Pattern Anal. Mach. Intell. *37*, 383–393.

Houmard, B., Small, C., Yang, L., Naluai-Cecchini, T., Cheng, E., Hassold, T., and Griswold, M. (2009). Global gene expression in the human fetal testis and ovary. Biol. Reprod. *81*, 438–443.

Iossifov, I., Levy, D., Allen, J., Ye, K., Ronemus, M., Lee, Y.H., Yamrom, B., and Wigler, M. (2015). Low load for disruptive mutations in autism genes and their biased transmission. Proc. Natl. Acad. Sci. USA *112*, E5600–E5607.

Kassambara, A., and Mundt, F. (2017). Factoextra: extract and visualize the results of multivariate data analyses.

Kosiol, C., Vinař, T., da Fonseca, R.R., Hubisz, M.J., Bustamante, C.D., Nielsen, R., and Siepel, A. (2008). Patterns of positive selection in six Mammalian genomes. PLoS Genet. *4*, e1000144.

Kumar, L., and Futschik, M.E. (2007). Mfuzz: a software package for soft clustering of microarray data. Bioinformation *2*, 5–7.

Lage, K., Hansen, N.T., Karlberg, E.O., Eklund, A.C., Roque, F.S., Donahoe, P.K., Szallasi, Z., Jensen, T.S., and Brunak, S. (2008). A large-scale analysis of tissue-specific pathology and gene expression of human disease genes and complexes. Proc. Natl. Acad. Sci. USA *105*, 20870–20875.

Langfelder, P., and Horvath, S. (2008). WGCNA: an R package for weighted correlation network analysis. BMC Bioinformatics *9*, 559.

Lein, E.S., Belgard, T.G., Hawrylycz, M., and Molnár, Z. (2017). Transcriptomic perspectives on neocortical structure, development, evolution, and disease. Annu. Rev. Neurosci. *40*, 629–652.

Lek, M., Karczewski, K.J., Minikel, E.V., Samocha, K.E., Banks, E., Fennell, T., O'Donnell-Luria, A.H., Ware, J.S., Hill, A.J., Cummings, B.B., et al.; Exome Aggregation Consortium (2016). Analysis of protein-coding genetic variation in 60,706 humans. Nature *536*, 285–291.

Li, M., Santpere, G., Kawasawa, Y.I., Evgrafov, O.V., Gulden, F.O., Pochareddy, S., Sunkin, S.M., Li, Z., Shin, Y., Zhu, Y., et al. (2018). Integrative functional genomic analysis of human brain development and neuropsychiatric risks. Science *362*, eaat7615.

Lonsdale, J., Thomas, J., Salvatore, M., Phillips, R., Lo, E., Shad, S., Hasz, R., Walters, G., Garcia, F., Young, N., et al.; GTEx Consortium (2013). The Genotype-Tissue Expression (GTEx) project. Nat. Genet. *45*, 580–585.

Love, M.I., Anders, S., and Huber, W. (2014). Differential analysis of count data: the DESeq2 package. Genome Biol. *15*, 10–1186.

Meehan, T.F., Conte, N., West, D.B., Jacobsen, J.O., Mason, J., Warren, J., Chen, C.K., Tudose, I., Relac, M., Matthews, P., et al.; International Mouse Phenotyping Consortium (2017). Disease model discovery from 3,328 gene knockouts by The International Mouse Phenotyping Consortium. Nat. Genet. *49*, 1231–1238.

Milinkovitch, M.C., Helaers, R., and Tzika, A.C. (2009). Historical constraints on vertebrate genome evolution. Genome Biol. Evol. *2*, 13–18.

Omer Javed, A., Li, Y., Muffat, J., Su, K.C., Cohen, M.A., Lungjangwa, T., Aubourg, P., Cheeseman, I.M., and Jaenisch, R. (2018). Microcephaly modeling of kinetochore mutation reveals a brain-specific phenotype. Cell Rep. *25*, 368–382.e5.

Pantalacci, S., and Semon, M. (2014). Transcriptomics of developing embryos and organs: a raising tool for EvoDevo. J. Exp. Zool. B Mol. Dev. Evol. *324*, 363–371.

R Core Team (2014). R: A language and environment for statistical computing (R Foundation for Statistical Computing).

Ripke, S., Neale, B.M., Corvin, A., Walters, J.T.R., Farh, K.H., Holmans, P.A., Lee, P., Bulik-Sullivan, B., Collier, D.A., Huang, H., et al.; Schizophrenia Working Group of the Psychiatric Genomics Consortium (2014). Biological insights from 108 schizophrenia-associated genetic loci. Nature *511*, 421–427.

Ruderfer, D.M., Hamamsy, T., Lek, M., Karczewski, K.J., Kavanagh, D., Samocha, K.E., Daly, M.J., MacArthur, D.G., Fromer, M., and Purcell, S.M.; Exome Aggregation Consortium (2016). Patterns of genic intolerance of rare copy number variation in 59,898 human exomes. Nat. Genet. *48*, 1107–1111.

Sarropoulos, I., Marin, R., Cardoso-Moreira, M., and Kaessmann, H. (2019). Developmental dynamics of lncRNAs across mammalian organs and species. Nature *571*, 510–514.

Satterstrom, F.K., Kosmicki, J.A., Wang, J., Breen, M.S., De Rubeis, S., An, J.Y., Peng, M., Collins, R., Grove, J., Klei, L., et al.; Autism Sequencing Consortium; iPSYCH-Broad Consortium (2020). Large-scale exome sequencing study implicates both developmental and functional changes in the neurobiology of autism. Cell *180*, 568–584.e23.

Shami, A.N., Zheng, X., Munyoki, S.K., Ma, Q., Manske, G.L., Green, C.D., Sukhwani, M., Orwig, K.E., Li, J.Z., and Hammoud, S.S. (2020). Single-cell RNA sequencing of human, macaque, and mouse testes uncovers conserved

CellPress
OPEN ACCESS

and divergent features of mammalian spermatogenesis. Dev. Cell *54*, 529–547.e12.

Shao, Y., Chen, C., Shen, H., He, B.Z., Yu, D., Jiang, S., Zhao, S., Gao, Z., Zhu, Z., Chen, X., et al. (2019).). GenTree, an integrated resource for analyzing the evolution and function of primate-specific coding genes. Genome Res. *29*, 682–696.

Si-Tayeb, K., Lemaigre, F.P., and Duncan, S.A. (2010). Organogenesis and development of the liver. Dev. Cell *18*, 175–189.

Sigalova, O.M., Shaeiri, A., Forneris, M., Furlong, E.E., and Zaugg, J.B. (2020). Predictive features of gene expression variation reveal mechanistic link with differential expression. Mol. Syst. Biol. *16*, e9539.

Silbereis, J.C., Pochareddy, S., Zhu, Y., Li, M., and Sestan, N. (2016). The cellular and molecular landscapes of the developing human central nervous system. Neuron *89*, 248–268.

Simonovsky, E., Schuster, R., and Yeger-Lotem, E. (2019). Large-scale analysis of human gene expression variability associates highly variable drug targets with lower drug effectiveness and safety. Bioinformatics *35*, 3028–3037.

Stenson, P.D., Mort, M., Ball, E.V., Evans, K., Hayden, M., Heywood, S., Hussain, M., Phillips, A.D., and Cooper, D.N. (2017). The Human Gene Mutation Database: towards a comprehensive repository of inherited mutation data for medical research, genetic diagnosis and next-generation sequencing studies. Hum. Genet. *136*, 665–677.

Stergachis, A.B., Neph, S., Sandstrom, R., Haugen, E., Reynolds, A.P., Zhang, M., Byron, R., Canfield, T., Stelhing-Sun, S., Lee, K., et al. (2014). Conservation of trans-acting circuitry during mammalian regulatory evolution. Nature *515*, 365–370.

Stoeger, T., Gerlach, M., Morimoto, R.I., and Nunes Amaral, L.A. (2018). Large-scale investigation of the reasons why potentially important genes are ignored. PLoS Biol. *16*, e2006643.

Ta-Shma, A., El-lahham, N., Edvardson, S., Stepensky, P., Nir, A., Perles, Z., Gavri, S., Golender, J., Yaakobi-Simhayoff, N., Shaag, A., et al. (2014). Conotruncal malformations and absent thymus due to a deleterious NKX2-6 mutation. J. Med. Genet. *51*, 268–270.

Vainio, S., and Lin, Y. (2002). Coordinating early kidney development: lessons from gene targeting. Nat. Rev. Genet. *3*, 533–543.

Verloes, A., Drunat, S., Gressens, P., and Passemard, S. (1993). Primary autosomal recessive microcephalies and seckel syndrome spectrum disorders. In GeneReviews, M.P. Adam, ed. (University of Washington, Seattle).

Wang, V.Y., and Zoghbi, H.Y. (2001). Genetic regulation of cerebellar development. Nat. Rev. Neurosci. *2*, 484–491.

Wang, J., Vasaikar, S., Shi, Z., Greer, M., and Zhang, B. (2017). WebGestalt 2017: a more comprehensive, powerful, flexible and interactive gene set enrichment analysis toolkit. Nucleic Acids Res. *45* (*W1*), W130–W137.

Wickham, H. (2007). Reshaping data with the reshape package. J. Stat. Softw. *21*, 1–20.

Wickham, H. (2009). ggplot2: Elegant Graphics for Data Analysis (Springer-Verlag New York).

Wickham, H. (2011). The split-apply-combine strategy for data analysis. J. Stat. Softw. *40*, 1–29.

Wickham, H. (2017).). tidyverse: easily install and load the "Tidyverse.".

Xue, Z., Huang, K., Cai, C., Cai, L., Jiang, C.Y., Feng, Y., Liu, Z., Zeng, Q., Cheng, L., Sun, Y.E., et al. (2013). Genetic programs in human and mouse early embryos revealed by single-cell RNA sequencing. Nature *500*, 593–597.

Yanai, I., Benjamin, H., Shmoish, M., Chalifa-Caspi, V., Shklar, M., Ophir, R., Bar-Even, A., Horn-Saban, S., Safran, M., Domany, E., et al. (2005). Genome-wide midrange transcription profiles reveal expression level relationships in human tissue specification. Bioinformatics *21*, 650–659.

Yates, A., Akanni, W., Amode, M.R., Barrell, D., Billis, K., Carvalho-Silva, D., Cummins, C., Clapham, P., Fitzgerald, S., Gil, L., et al. (2016). Ensembl 2016. Nucleic Acids Res. *44* (*D1*), D710–D716.

Zhang, Y.E., Landback, P., Vibranovski, M., and Long, M. (2012). New genes expressed in human brains: implications for annotating evolving genomes. BioEssays *34*, 982–991.

Zhang, H.M., Liu, T., Liu, C.J., Song, S., Zhang, X., Liu, W., Jia, H., Xue, Y., and Guo, A.Y. (2015). AnimalTFDB 2.0: a resource for expression, prediction and functional study of animal transcription factors. Nucleic Acids Res. *43*, D76–D81.

Zhu, Y., Sousa, A.M.M., Gao, T., Skarica, M., Li, M., Santpere, G., Esteller-Cucala, P., Juan, D., Ferrández-Peral, L., Gulden, F.O., et al. (2018). Spatiotemporal transcriptomic divergence across human and macaque brain development. Science *362*.

STAR★METHODS

KEY RESOURCES TABLE

REAGENT or RESOURCE	SOURCE	IDENTIFIER
Deposited Data		
human developmental time-series for the brain, cerebellum, heart, kidney, liver, ovary and testis	Cardoso-Moreira et al., 2019	ArrayExpress: E-MTAB-6814
rhesus macaque developmental time-series for the brain, cerebellum, heart, kidney, liver, ovary and testis	Cardoso-Moreira et al., 2019	ArrayExpress: E-MTAB-6813
mouse developmental time-series for the brain, cerebellum, heart, kidney, liver, ovary and testis	Cardoso-Moreira et al., 2019	ArrayExpress: E-MTAB-6798
rat developmental time-series for the brain, cerebellum, heart, kidney, liver, ovary and testis	Cardoso-Moreira et al., 2019	ArrayExpress: E-MTAB-6811
rabbit developmental time-series for the brain, cerebellum, heart, kidney, liver, ovary and testis	Cardoso-Moreira et al., 2019	ArrayExpress: E-MTAB-6782
Software and Algorithms		
WGCNA (1.61)	Langfelder and Horvath, 2008	https://horvath.genetics.ucla.edu/html/CoexpressionNetwork/Rpackages/WGCNA/
DESeq2 (1.12.4)	Love et al., 2014	https://bioconductor.org/packages/release/bioc/html/DESeq2.html
WebGestalt (0.0.5)	Wang et al., 2017	https://cran.r-project.org/web/packages/WebGestaltR/index.html
mFuzz (2.32.0)	Futschik and Carlisle, 2005; Kumar and Futschik, 2007	https://www.bioconductor.org/packages/release/bioc/html/Mfuzz.html
GPClust	Hensman et al., 2012, 2013, 2015	https://github.com/SheffieldML/GPclust
R (3.3.2)	R Core Team, 2014	https://www.r-project.org/
ggplot2 (2.2.1)	Wickham, 2009	https://cran.r-project.org/web/packages/ggplot2/index.html
gridExtra (2.2.1)	Auguie, 2016	https://cran.r-project.org/web/packages/gridExtra/index.html
reshape2 (1.4.2)	Wickham, 2007	https://cran.r-project.org/web/packages/reshape2/index.html
plyr (1.8.4)	Wickham, 2011	https://cran.r-project.org/web/packages/plyr/index.html
factoextra (1.0.4)	Kassambara and Mundt, 2017	https://cran.r-project.org/web/packages/factoextra/index.html
tidyverse (1.2.1)	Wickham, 2017	https://cran.r-project.org/web/packages/tidyverse/index.html

RESOURCE AVAILABILITY

Lead contact

Further information and requests for resources should be directed to and will be fulfilled by the Lead Contact, Margarida Cardoso-Moreira (margarida.cardosomoreira@crick.ac.uk).

Materials availability

This study did not generate new unique reagents.

Data and code availability

This study did not generate any unique datasets or code.

METHOD DETAILS

Resource

From a mammalian resource on organ development (Cardoso-Moreira et al., 2019), we analyzed data from 1,443 strand-specific RNA-seq libraries sequenced to a median depth of 33 million reads: 297 from human, 316 from mouse (outbred strain CD-1 - RjOrl:SWISS), 350 from rat (outbred strain Holtzman SD), 315 from rabbit (outbred New Zealand breed) and 165 from rhesus macaque. The organs, developmental stages and replicates sampled in each species are described in Table S4. The mouse time series started at e10.5 and there were prenatal samples available for each day until birth (i.e., e18.5). There were postnatal samples for 5 stages: P0, P3, P14, P28 and P63. The rat time series started at e11 and there were prenatal samples available for each day until birth (i.e., e20). There were postnatal samples for 6 stages: P0, P3, P7, P14, P42 and P112. The rabbit time series started at e12 and there were 11 prenatal stages available up to and until e27 (gestation lasts ~29-32 days). There were postnatal samples for 4 stages: P0, P14, P84 and P186-P548. Finally, the time series for rhesus macaque started at a late fetal stage (e93) and there were 5 prenatal stages available up to and until e130 (gestation last ~167 days). There were postnatal samples for 8 stages: P0, P23, 5-6 months of age, 1 year, 3 years, 9 years, 14-15 years, and 20-26 years. For mouse, rat, and rabbit, there were typically 4 replicates (2 males and 2 females) per stage, except for ovary and testis (2 replicates). For human and rhesus macaque, the median number of replicates was 2.

Gene co-expression networks

We built gene co-expression networks using weighted correlation network analysis (WGCNA 1.61) (Langfelder and Horvath, 2008). We used as input data the read counts after applying the variance stabilizing (VS) transformation implemented in DESeq2 (1.12.4) (Love et al., 2014). Each stage was represented by the median across replicates. In addition to protein-coding genes, we included a set of 5,887 lncRNAs that show significant differential temporal expression in at least one organ and that show multiple signatures for being enriched with functional genes (Sarropoulos et al., 2019). We only excluded genes that failed to reach an RPKM (reads per kilobase of exon model per million mapped reads) across all stages and organs higher than 1. Using WGCNA we built a signed network (based on the correlation across all stages and organs) using a power of 10 and default parameters. We then correlated the eigengenes for each module with the sample traits (i.e., organ and developmental stage).

We characterized each module in terms of biological processes and disease enrichments (GLAD4U) using the R implementation of WebGestalt (FDR $\leq$ 0.01; version 0.0.5) (Wang et al., 2017). The lists of TFs are from the animalTFDB (version 2.0) (Zhang et al., 2015) and the list of RNA-binding proteins are from the work of Gerstberger and colleagues (Gerstberger et al., 2014).

Inherited disease genes

The list of genes associated with human inherited disease was obtained from the manually curated HGMD (PRO 17.1) (Stenson et al., 2017). We only used genes with disease-causing mutations (DM tag; Table S2). Genes associated with DM mutations were mapped onto the Unified Medical Language System (UMLS), and aggregated into one or more of the following high level disease types: Eye, Nervous system, Reproductive, Cancer, Skin, Heart, Blood, Blood Coagulation, Endocrine, Immune, Digestive, Genitourinary, Metabolic, Ear Nose & Throat, Respiratory, Developmental, Musculoskeletal, and Psychiatric (Stenson et al., 2017).

We also characterized the developmental profiles of genes associated with three neurodevelopmental disorders: primary microcephaly, autism spectrum disorders and schizophrenia. For all three disorders we limited our analyses to those genes with dynamic temporal expression in the brain and asked if they were enriched in particular clusters when compared to all genes showing dynamic temporal expression in the brain (binomial tests with Bonferroni correction). This translated into 15 genes associated with primary microcephaly and with dynamic temporal profiles in the brain (out of a set of 16 genes associated with this condition; Verloes et al., 1993), 79 genes associated with autism spectrum disorders (out of 102; Satterstrom et al., 2020) and 45 genes associated with schizophrenia (out of 75; Ripke et al., 2014). For our analysis of genes associated with schizophrenia we only considered loci where at most two genes were associated with the causative variant. We also performed the analysis of genes associated with autism spectrum disorders using a larger dataset of autism associated genes (164 with dynamic temporal profiles out of 233; Iossifov et al., 2015) and obtained the same result (i.e., significant enrichment in cluster 8, 62 out of 164 genes, Bonferroni-corrected P-value = 8×10^{-9}). The list of human essential genes was obtained from the work of Bartha and colleagues (Bartha et al., 2018).

The time- and organ-specificity indexes were based on the Tau metric of tissue-specificity (Yanai et al., 2005) and were retrieved from the developmental resource (Cardoso-Moreira et al., 2019). Both indexes range from 0 (broad expression) to 1 (restricted expression). The pleiotropy index is the number of samples where a gene is expressed (RPKM > 1) over the total number of samples.

The most common temporal profiles in each organ were identified using the soft-clustering approach (c-means) implemented in the R package mFuzz (2.32.0) (Futschik and Carlisle, 2005; Kumar and Futschik, 2007). The clustering was restricted to genes previously identified as showing significant temporal differential expression in each organ (i.e., developmentally dynamic genes) (Cardoso-Moreira et al., 2019). We used as input the VS-transformed counts. Prior to clustering, mFuzz standardizes the expression values of every gene so that the average expression value for each gene is zero and the standard deviation of its expression profile is one. This is done to make genes comparable. The number of clusters was set to 6-8 depending on the organ.

Age of human genes and orthology

The classification of human genes according to their evolutionary age (i.e., to when they first originated) was retrived from the GenTree database (http://gentree.ioz.ac.cn/) (Shao et al., 2019). The age assignments are based on the human genome assembly hg19 and on Ensembl version 73 annotations.

The lists of orthologs between human genes and mouse, rat, rabbit, and rhesus macaque was obtained using Ensembl's BioMart (Yates et al., 2016). The lists of orthologs are based on Ensembl version 85 annotations.

Organ developmental trajectories

For each organ, we compared the developmental trajectories of orthologous genes previously identified as showing significant temporal differential expression (Cardoso-Moreira et al., 2019). We used as input the VS-transformed counts (median across replicates) for matching stages between human and each of the other species. The developmental stage correspondences across species were retrieved from the developmental resource (Cardoso-Moreira et al., 2019). We used GPClust (Hensman et al., 2012, 2013, 2015), which clusters time-series using Gaussian processes, to cluster the combined data for human and each of the other species. We set the noise variance (k2.variance.fix) to 0.7 and let GPClust infer the number of clusters. For each gene, GPClust assigned the probability of it belonging to each of the clusters. Therefore, for each gene we obtained a vector of probabilities that could be directly compared between pairs of 1:1 orthologs. We calculated the probability that pairs of orthologs were in the same cluster and used an FDR cut off of 5% to identify the genes that differed in trajectory between human and each of the other species. In Table S3, we provide the *P*-values for each organ and species (adjusted for multiple testing using the Benjamini-Hochberg procedure (Benjamini and Hochberg, 1995)) for the null hypothesis that orthologs have the same trajectory, and their classification as 'same' or 'different' based on an FDR of 5%.

Changing the noise variance (k2.variance.fix) impacts the number of clusters that are identified. The fewer the clusters, the more distinct are the expression profiles among the clusters, and vice versa. The degree of distinctiveness among the clusters impacts the type of trajectory differences that are identified between species. If the k2.variance.fix is increased to 1, the number of clusters identified decreases and only genes with opposing developmental trajectories (i.e., whose expression is negatively correlated throughout the time series) are identified as having trajectory differences between species (Figure S7D). Decreasing the k2.variance.fix to 0.5 has the opposite effect; a larger number of clusters are identified and a larger number of genes with subtler temporal differences is identified (Figure S7D). In this study we were interested both in genes with opposing developmental trajectories (e.g., *RIT1* and *ABCA1* in Figure 6B) and in genes that differ in only part of the time series (e.g., *CLP1* and *ABCB4* in Figure 6B). A k2.variance.fix of 1 identified the former but a k2.variance.fix of 0.7 was required to identify the latter (all genes identified using a k2.variance.fix of 1 are also identified using the 0.7 cutoff). Further decreasing the k2.variance.fix increases the number of clusters but the extra clusters identified are not enriched with specific biological processes and are strongly biased toward having genes from only one of the species (data not shown). For these reasons, we decided to use a k2.variance.fix of 0.7 in our work. However, in Table S5 we provide the results from this analysis (Benjamini-Hochberg adjusted *P*-values) using the three cutoffs (1, 0.7 and 0.5). Irrespective of the k2.variance.fix threshold used, we always observe more differences between human and mouse (and rat) than between human and rabbit (Figure S6D), and the correlation coefficient distributions for genes identified as having different trajectories are at least as low as those of genes that have no orthology relationship with each other (Figure S7D).

Characterization trajectory differences

The PsychENCODE consortium provides calls of differential gene expression between human and rhesus macaque for 16 brain regions (11 areas of the cerebral neocortex, hippocampus, amygdala, striatum, mediodorsal nucleus of thalamus, and cerebellar cortex) for 3 developmental periods (prenatal, postnatal, and adult) (Zhu et al., 2018). We compared the genes that we identified as having similar or different brain developmental trajectories between human and rhesus macaque in terms of the number of comparisons (regions * developmental periods) that the PsychENCODE dataset called as differentially expressed between the two species. We did this analysis using 1) the set of 11 cerebral neocortex samples (Figure S6B), and 2) all brain regions except for the cerebellum (i.e., 15 regions). The result was the same.

We calculated variation in gene expression across the GTEx dataset (Lonsdale et al., 2013) using three measures: 1) the standard deviation (SD), 2) the coefficient of variation (CV, standard deviation divided by the mean), and 3) the residual CV. The SD and CV are the classical measures to estimate variation in gene expression but have known biases: SD tends to be biased toward genes with high expression levels, whereas the CV tends to be biased toward genes with low expression levels (Simonovsky et al., 2019). Because expression variation is highly correlated with the levels of gene expression (Anders and Huber, 2010), we also used a measure of expression variation that takes into account gene expression levels, the residual CV (Sigalova et al., 2020). The residual CV uses the residuals from a locally weighed regression (LOESS) of the CV on median expression, and it is highly correlated with other measures of expression variation that take into account expression levels (Sigalova et al., 2020). Using all three measures, we consistently found that in the brain and testis, genes with trajectory differences tend to show less variation in gene expression than genes with similar trajectories, whereas no differences are observed in the other organs. It is unclear why there is a difference for the brain and testis. The values for CV and residual CV shown in Figure S6E are from Sigalova and colleagues (Sigalova et al., 2020) based on GTEx samples for the cortex (matched to our brain samples), cerebellum, left ventricle (matched to our heart samples, similar results using the atrial appendage samples), liver and testis.

We compared genes with similar and different organ trajectories using two different metrics of functional constraint: 1) the residual variation intolerance score (RVIS), and 2) the probability of being intolerant to loss-of-function mutations (pLI score). Both metrics were applied to data from the Exome Aggregation Consortium (ExAC) (Lek et al., 2016). We obtained the pLI and RVIS scores from the work of Dickinson and colleagues (Dickinson et al., 2016). The RVIS and pLI scores give similar results. We used the copy-number variation (CNV) intolerance score as applied to the ExAC data from the work of Ruderfer and colleagues (Ruderfer et al., 2016).

The animal and organ silhouettes used in the figures were originally published by Cardoso-Moreira and colleagues (Cardoso-Moreira et al., 2019).

QUANTIFICATION AND STATISTICAL ANALYSIS

Statistical analyses and plots were done in R (3.3.2) (R Core Team, 2014). Plots were created using the R packages ggplot2 (2.2.1) (Wickham, 2009), gridExtra (2.2.1) (Auguie, 2016), reshape2 (1.4.2) (Wickham, 2007), plyr (1.8.4) (Wickham, 2011), factoextra (1.0.4) (Kassambara and Mundt, 2017), and tidyverse (1.2.1) (Wickham, 2017).

The statistical details of our analyses are reported in the figure legends, figures, Results and STAR Methods. These include the statistical tests used, the exact numbers of genes tested and the multiple-test corrections performed.

Cell Reports, Volume *33*

Supplemental Information

Developmental Gene Expression Differences between Humans and Mammalian Models

Margarida Cardoso-Moreira, Ioannis Sarropoulos, Britta Velten, Matthew Mort, David N. Cooper, Wolfgang Huber, and Henrik Kaessmann

Figure S1

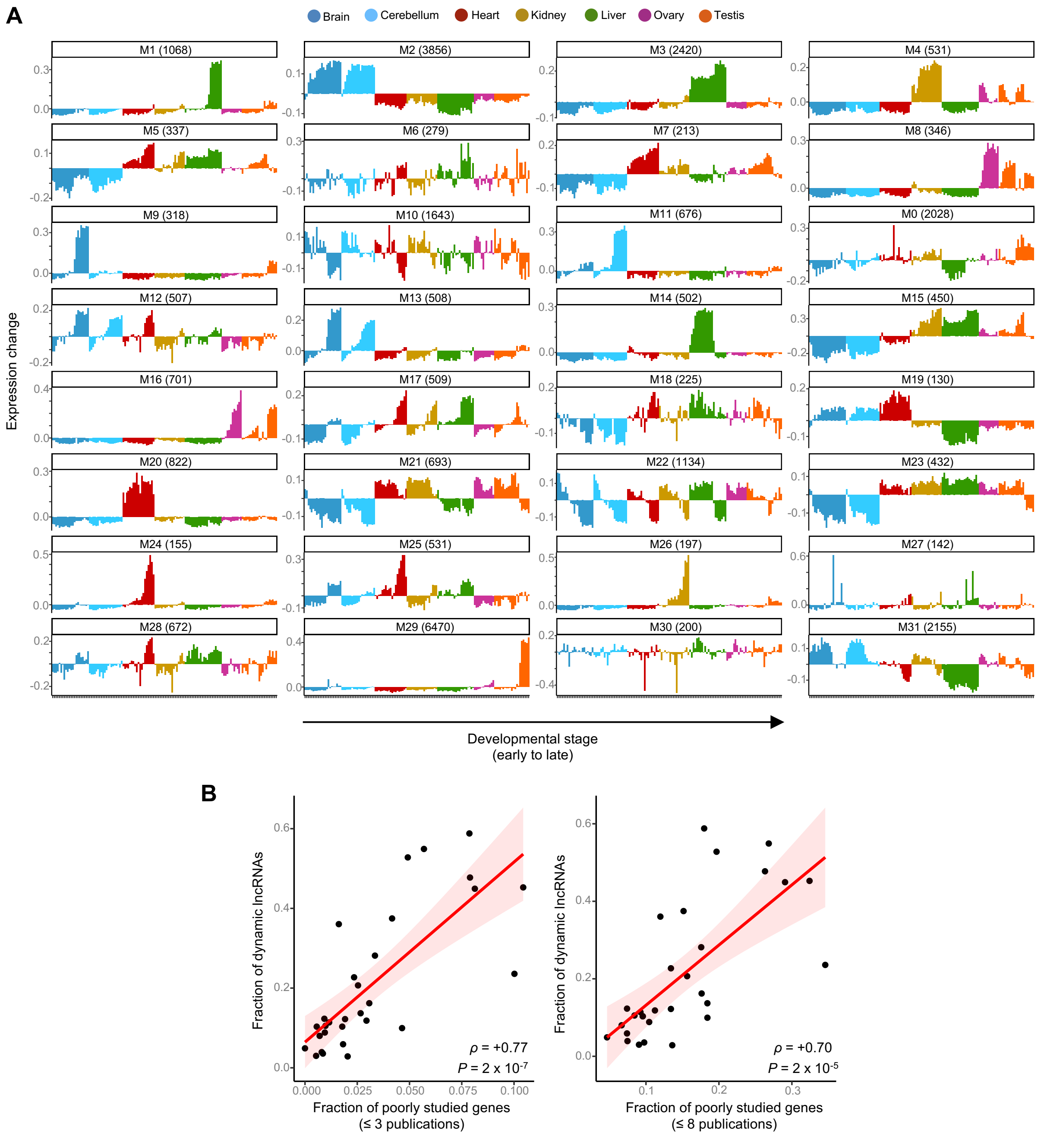

Figure S1. Human weighted gene co-expression network, Related to Figure 1 (A) Organ developmental profiles for each module; shown is the module's eigengene. **(B)** There is a strong positive correlation between the fraction of developmentally dynamic lncRNAs in a module and the fraction of poorly studied protein-coding genes. Poorly studied genes are those with 3 or fewer publications (left) or those with 8 or fewer publications (right). Data on the number of publications are from Stoeger and colleagues (Stoeger et al., 2018). Shaded area corresponds to the 95% confidence interval.

Figure S2

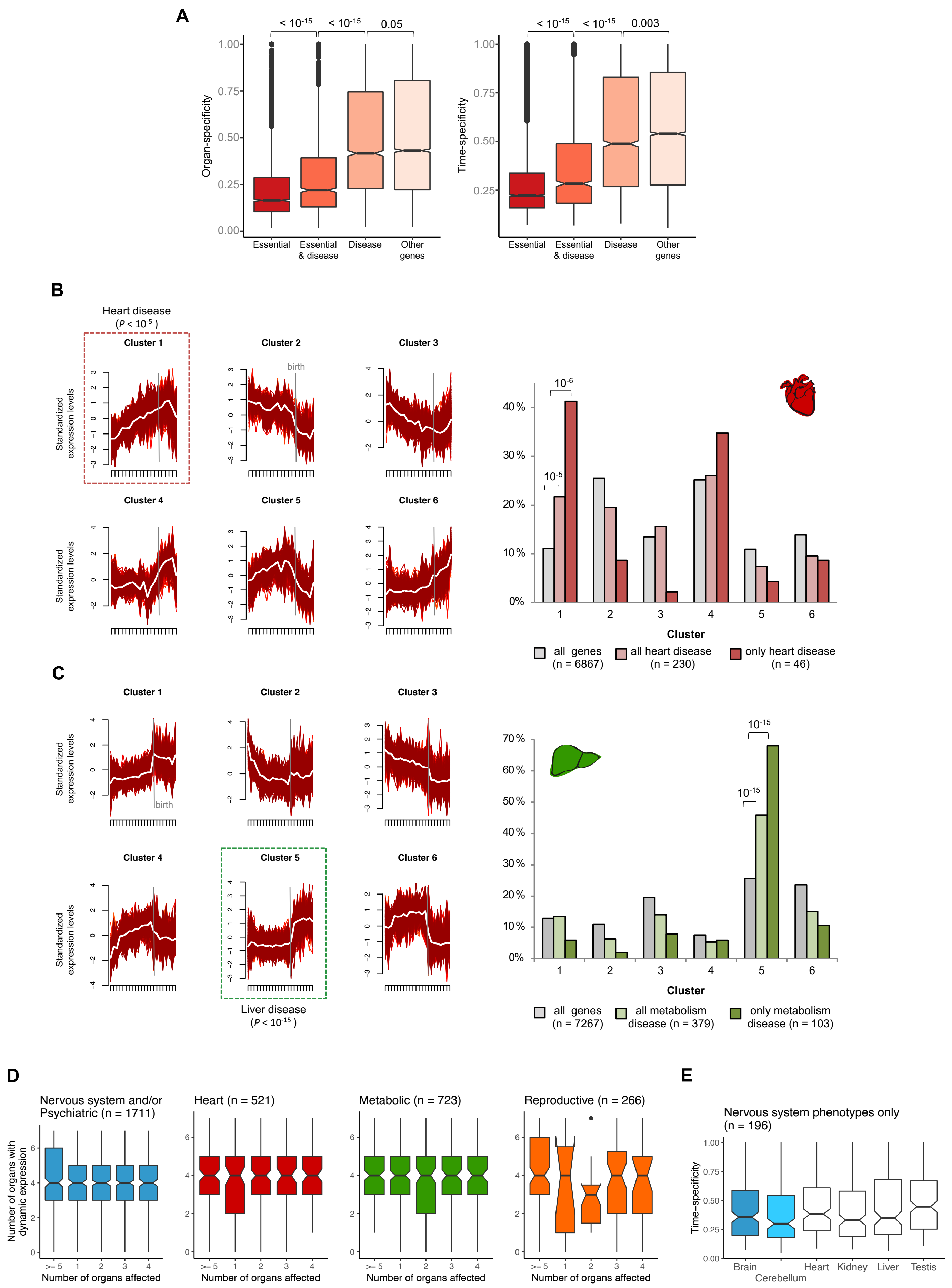
A
Organ-specificity
Time-specificity
Essential
Essential & disease
Disease
Other genes
< 10-15
0.05
0.003
B
Heart disease (P < 10-5)
Cluster 1
Cluster 2
Cluster 3
Cluster 4
Cluster 5
Cluster 6
birth
Standardized expression levels
10-6
10-5
Cluster
all genes (n = 6867)
all heart disease (n = 230)
only heart disease (n = 46)
C
Liver disease (P < 10-15)
10-15
all genes (n = 7267)
all metabolism disease (n = 379)
only metabolism disease (n = 103)
D
Nervous system and/or Psychiatric (n = 1711)
Heart (n = 521)
Metabolic (n = 723)
Reproductive (n = 266)
Number of organs with dynamic expression
Number of organs affected
E
Nervous system phenotypes only (n = 196)
Brain
Cerebellum
Heart
Kidney
Liver
Testis

Figure S2. Spatiotemporal profiles of disease genes, Related to Figure 2. (A) Organ- and time-specificity (median across organs) for genes in different classes of phenotypic severity (*P*-values from Wilcoxon rank sum test, two-sided). The box plots depict the median ± 25th and 75th percentiles, whiskers at 1.5 times the interquartile range. The number of genes in each class is provided in Figure 2A. **(B)** Distribution of genes associated with heart disease among the 6 heart clusters. The developmental profiles of the 6 clusters are shown on the left. The y-axis shows standardized expression levels (Methods). The profile of each gene in the cluster is shown in red; the white line shows the cluster center. Cluster 1 is enriched for heart disease-associated genes both when using all genes associated with a heart phenotype (50 out of 230 genes) and when restricting the set to those exclusively associated with the heart (19 out of 46 genes) (*P*-values from binomial tests after Bonferroni correction). **(C)** Distribution of genes associated with metabolic diseases among the 6 liver clusters. The developmental profiles of the 6 clusters are shown on the left. The y-axis shows standardized expression levels (Methods). The profile of each gene in the cluster is shown in red; the white line shows the cluster centre. Cluster 5 is enriched for metabolic disease-associated genes both when using all genes associated with a metabolic phenotype (174 out of 379 genes) and when restricting the set to those exclusively associated with metabolism (70 out of 103 genes) (*P*-values from binomial tests after Bonferroni correction). **(D)** Number of organs where genes have dynamic temporal profiles as a function of the number of organs where they are known to cause disease. Number of genes in each disease class in parenthesis. **(E)** Time-specificity in different organs for genes associated exclusively with nervous system phenotypes (n = 196 genes). In **(D)** and **(E)** the box plots depict the median ± 25th and 75th percentiles, whiskers at 1.5 times the interquartile range.

Figure S3

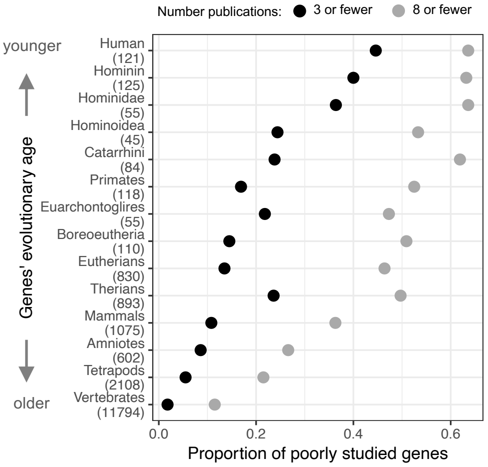

Figure S3: Recently originated genes tend to be poorly studied, Related to Figure 3. The proportion of poorly studied genes (i.e., those with 3 or fewer publications (black) or those with 8 or fewer publications (grey)) increases for genes of successively younger evolutionary ages (i.e. with more recent origins). In this analysis, the youngest genes are those that are human-specific (top) and the oldest are those shared across vertebrates (bottom). In parenthesis are the number of genes in each age class. Data on the evolutionary age of genes are from GenTree (Shao et al., 2019) and data on the number of publications are from Stoeger and colleagues (Stoeger et al., 2018). The dataset of Stoeger and colleagues does not include data for most recently originated genes (those originated along the primate lineage), so the estimates provided are conservative (i.e., current knowledge on these genes is even more limited).

Figure S4

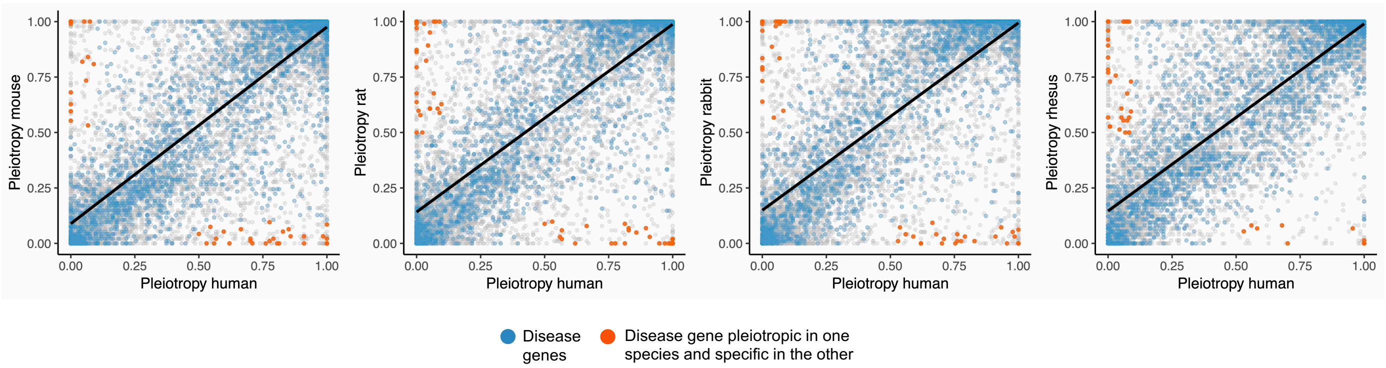

Figure S4: Breadth of spatiotemporal expression of human genes and their orthologs in mouse, rat, rabbit and rhesus, Related to Figure 4. Relationship between human expression pleiotropy and those of the other species. The blue dots denote disease-associated genes and the orange dots denote disease-associated genes expressed in at least 50% of the samples in one species but in less than 10% of the samples in the other. Note that the rhesus macaque time series is shorter, not covering the period of embryonic development sampled in the other species.

Figure S5

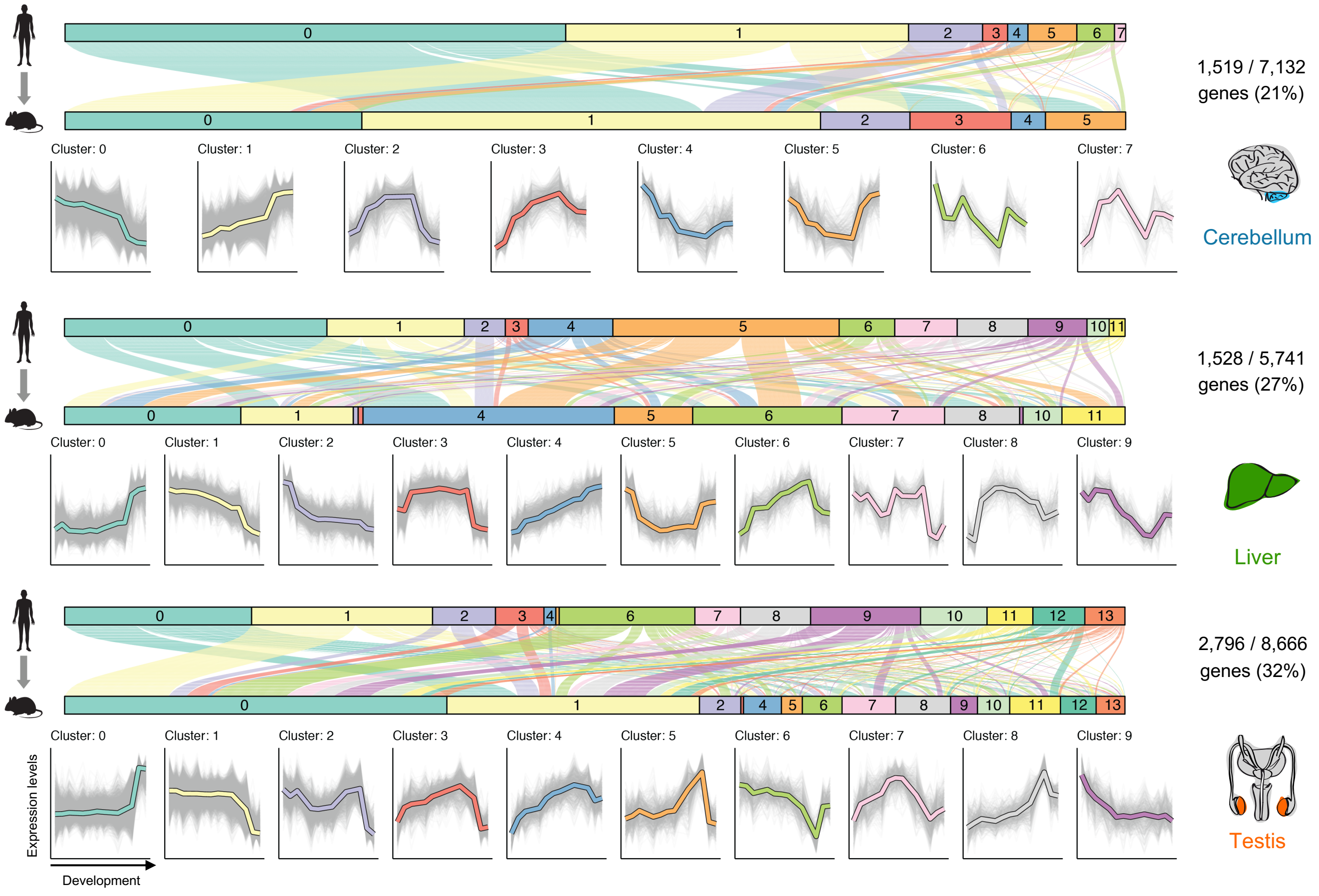

Figure S5: Developmental trajectory differences between human and mouse in cerebellum, liver and testis, Related to Figure 5. We used soft clustering to group human genes and their mouse orthologs according to their temporal expression in each organ. The genes for which the human and mouse ortholog were assigned to a different cluster are shown on top. Each line represents a pair of human-mouse orthologs and shows the cluster assignment in human (top) and mouse (bottom). The lines are colored according to the human cluster assignment. The clusters identified in each organ are shown below (grey lines correspond to individual genes and the colored lines to the cluster center, which is akin to the median expression of the cluster). The y-axis shows the log normalized expression levels and the x-axis shows the samples ordered from early to late development. In liver and testis, only the profiles of the first 9 clusters are shown. This panel complements Figure 5 which shows the same analysis for brain, heart and kidney.

Figure S6

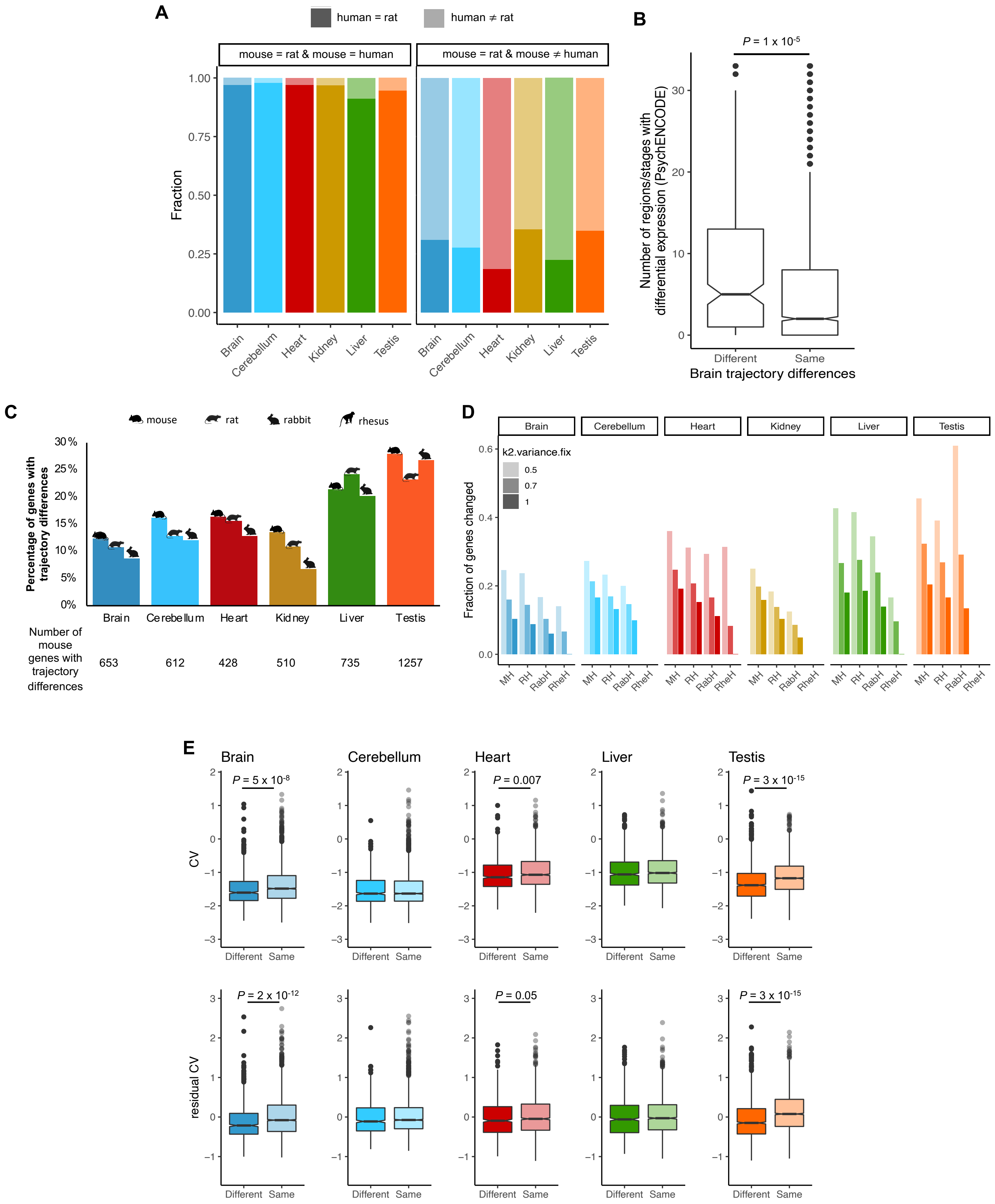

A
human = rat
human ≠ rat
mouse = rat & mouse = human
mouse = rat & mouse ≠ human
Fraction
1.00
0.75
0.50
0.25
0.00
Brain
Cerebellum
Heart
Kidney
Liver
Testis
B
P = 1 x 10-5
Number of regions/stages with differential expression (PsychENCODE)
Different
Same
Brain trajectory differences
C
mouse
rat
rabbit
rhesus
Percentage of genes with trajectory differences
30%
25%
20%
15%
10%
5%
0%
Number of mouse genes with trajectory differences
653
612
428
510
735
1257
D
k2.variance.fix
0.5
0.7
1
Fraction of genes changed
MH
RH
RabH
RheH
E
CV
residual CV
P = 5 x 10-8
P = 0.007
P = 3 x 10-15
P = 2 x 10-12
P = 0.05
P = 3 x 10-15

Figure S6: Developmental trajectory differences between human and the other species, Related to Figure 6. (A) If a gene has a similar trajectory between mouse and rat, then the human-mouse comparison is expected to give a similar result to the human-rat comparison. The panel on the left shows that most genes (96% across organs) called as having a similar trajectory between mouse and rat and between human and mouse, are also called as having a similar trajectory between human and rat (as expected). The panel on the right shows that most genes (70% across organs) called as having a similar trajectory between mouse and rat and a different trajectory between human and mouse, are also called as having a different trajectory between human and rat. **(B)** The PsychENCODE consortium compared gene expression profiles between human and rhesus macaque for 11 regions of the neocortex and for 3 developmental periods (i.e., prenatal, postnatal, and adult), for a total of 33 comparisons. We compared the genes that we identified as having similar or different brain developmental trajectories between human and rhesus macaque in terms of the number of comparisons that the PsychENCODE dataset called as differentially expressed. The PsychENCODE dataset provided comparisons for 234 of the 399 genes that we identified as having trajectory differences and for 2,327 of the 5,615 genes that we identified as having similar trajectories. The *P*-value is from a Wilcoxon rank sum test. The box plots depict the median ± 25th and 75th percentiles, whiskers at 1.5 times the interquartile range. **(C)** Percentage of genes in each organ that have different trajectories between human and mouse, rat and rabbit. This analysis is similar to that in Figure 6A except that the set of genes tested for trajectory differences is the same for all species (i.e., 5287 1:1 orthologous genes across the four species tested in the brain, 3786 in the cerebellum, 2608 in the heart, 3767 in the kidney, 3430 in the liver and 4500 in the testis). **(D)** Proportion of genes with different trajectories between human (H) and mouse (M), rat (R), rabbit (Rab) and Rhesus (Rhe) using different thresholds of k2.variance.fix (k = 1, k = 0.7 and k = 0.5). The set of genes identified using k=0.7 includes the set of genes identified using k=1; the set of genes identified using k=0.5 includes the sets of genes identified using k=1 and k=0.7. **(E)** Differences in gene expression variation among humans (GTEx dataset) between genes identified as having different or similar trajectories in the human-mouse comparison. The top row shows the variation of gene expression estimated as the coefficient of variation (CV; standard deviation divided by the mean) and the bottom row shows a derived measure of the coefficient of variation, residual CV, which also takes into consideration differences in gene expression levels (Methods). All *P*-values are from Wilcoxon rank sum tests after Bonferroni correction. The box plots depict the median ± 25th and 75th percentiles, whiskers at 1.5 times the interquartile range.

Figure S7

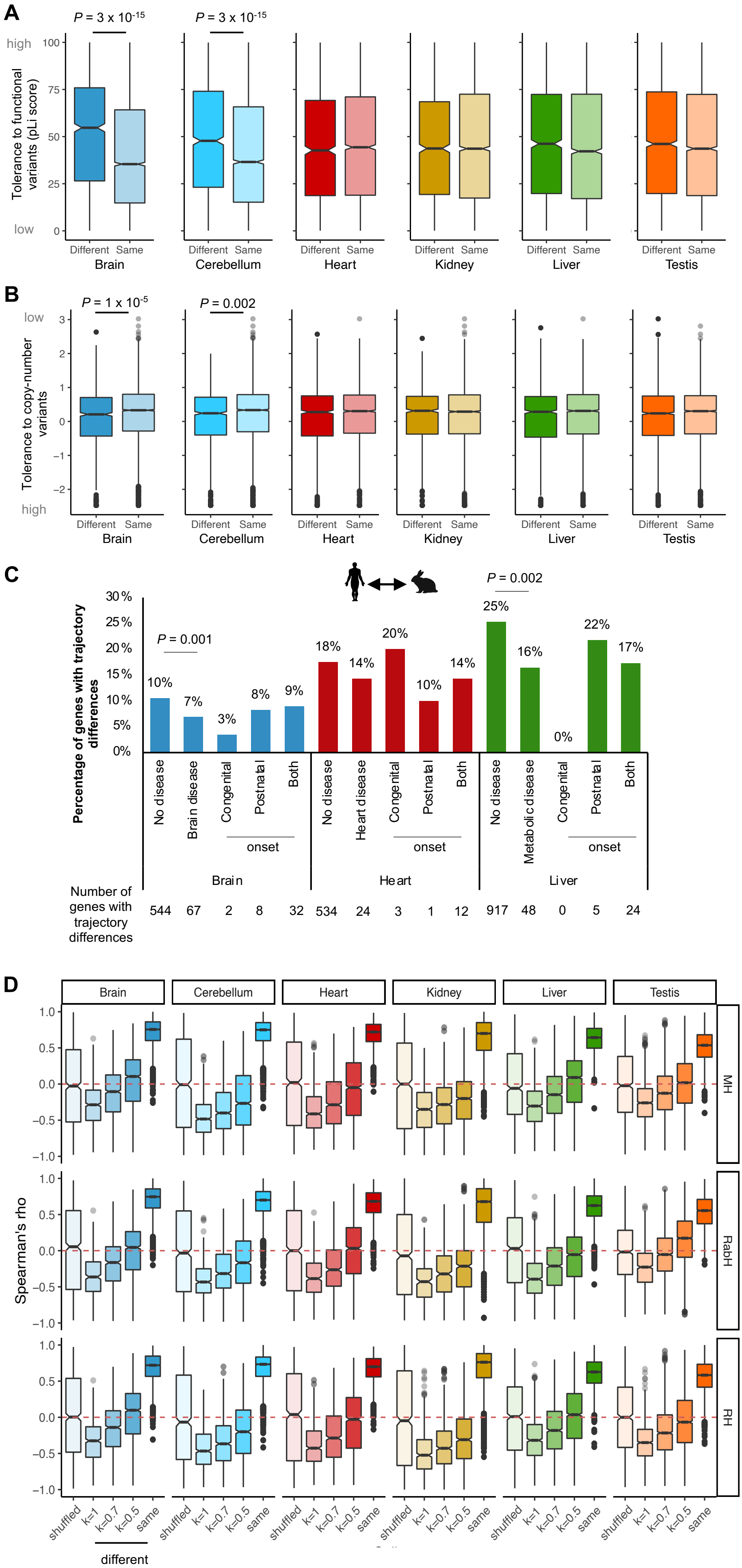

A
P = 3 x 10-15
P = 3 x 10-15
high
low
Tolerance to functional variants (pLi score)
100
75
50
25
0
Different Same
Brain
Cerebellum
Heart
Kidney
Liver
Testis
B
P = 1 x 10-5
P = 0.002
low
high
Tolerance to copy-number variants
3
2
1
0
−1
−2
C
P = 0.001
P = 0.002
Percentage of genes with trajectory differences
30%
25%
20%
15%
10%
5%
0%
10%
7%
3%
8%
9%
18%
14%
20%
10%
14%
25%
16%
0%
22%
17%
No disease
Brain disease
Congenital
Postnatal
Both
onset
Heart disease
Metabolic disease
Brain
Heart
Liver
Number of genes with trajectory differences
544 67 2 8 32 534 24 3 1 12 917 48 0 5 24
D
Brain
Cerebellum
Heart
Kidney
Liver
Testis
MH
RabH
RH
Spearman's rho
1.0
0.5
0.0
−0.5
−1.0
shuffled
k=1
k=0.7
k=0.5
same
different

Figure S7: Developmental trajectory differences between human and other species, Related to Figure 6. (A) Intolerance to functional mutations (probability that a gene is intolerant to a loss-of-function mutation, known as 'pLI' score) for genes with different or similar trajectories between human and mouse. Low values on the y-axis means low tolerance to functional mutations. **(B)** Intolerance to copy-number variants (probability that a gene is intolerant duplication and/or deletion polymorphisms) for genes with different or similar trajectories between human and mouse. Low values on the y-axis means high tolerance to copy-number variants. **(C)** Percentage of genes in brain, heart and liver that differ in trajectories between human and rabbit. Bonferroni-corrected *P*-values for comparisons between disease and non-disease genes are from Fisher's exact tests and Bonferroni-corrected *P*-values for comparisons of disease genes with different ages of onset are from binomial tests. **(D)** Spearman correlation of the temporal expression of human genes and their orthologs in mouse (top), rabbit (middle) and rat (bottom) for genes called as having different trajectories using different values of k2.variance.fix (k = 1, k = 0.7 and k = 0.5) and for genes called as having the same trajectory. Correlations for unrelated (non-orthologous) pairs of genes are also shown ("shuffled") as reference.
